# Artemether Alleviates Diabetic Kidney Disease by Modulating Amino Acid Metabolism

**DOI:** 10.1155/2022/7339611

**Published:** 2022-05-11

**Authors:** Guangli Rong, Wenci Weng, Jingting Huang, Yijun Chen, Xuewen Yu, Rui Yuan, Xiufen Gu, Xia Wu, Yuchun Cai, Pengxun Han, Mumin Shao, Huili Sun, Na Ge

**Affiliations:** ^1^Department of Nephrology, The Fourth Clinical Medical School of Guangzhou University of Chinese Medicine, Shenzhen Traditional Chinese Medicine Hospital, Shenzhen, 518033 Guangdong, China; ^2^Department of Pathology, The Fourth Clinical Medical School of Guangzhou University of Chinese Medicine, Shenzhen Traditional Chinese Medicine Hospital, Shenzhen, 518033 Guangdong, China; ^3^Department of Clinical Laboratory, The Fourth Clinical Medical School of Guangzhou University of Chinese Medicine, Shenzhen Traditional Chinese Medicine Hospital, Shenzhen, 518033 Guangdong, China

## Abstract

Diabetes is a worldwide metabolic disease with rapid growing incidence, characterized by hyperglycemia. Diabetic kidney disease (DKD), the leading cause of chronic kidney disease (CKD), has a high morbidity according to the constantly increasing diabetic patients and always develops irreversible deterioration of renal function. Though different in pathogenesis, clinical manifestations, and therapies, both type 1 diabetes mellitus (T1DM) and type 2 diabetes mellitus (T2DM) can evolve into DKD. Since amino acids are both biomarkers and causal agents, rarely report has been made about its metabolism which lies in T1DM- and T2DM-related kidney disease. This study was designed to investigate artemether in adjusting renal amino acid metabolism in T1DM and T2DM mice. Artemether was applied as treatment in streptozotocin (STZ) induced T1DM mice and db/db T2DM mice, respectively. Artemether-treated mice showed lower FBG and HbA1c and reduced urinary albumin excretion, as well as urinary NAG. Both types of diabetic mice showed enlarged kidneys, as confirmed by increased kidney weight and the ratio of kidney weight to body weight. Artemether normalized kidney size and thus attenuated renal hypertrophy. Kidney tissue UPLC-MS analysis showed that branched-chain amino acids (BCAAs) and citrulline were upregulated in diabetic mice without treatment and downregulated after being treated with artemether. Expressions of glutamine, glutamic acid, aspartic acid, ornithine, glycine, histidine, phenylalanine and threonine were decreased in both types of diabetic mice whereas they increased after artemether treatment. The study demonstrates the initial evidence that artemether exerted renal protection in DKD by modulating amino acid metabolism.

## 1. Introduction

Approximately 40% of diabetic patients suffer from DKD, the leading cause of CKD in the world [1, 2]. Studies from China indicate that nearly 21.3% of the participants with diabetes have CKD [3, 4]. DKD always develops irreversible deterioration of renal function [5]. Therefore, further deciphering the pathophysiological mechanism of DKD and finding potential intervention are in a strong clinical need.

The interactions between metabolic disorders and DKD are immensely sophisticated. Complicated progressive metabolic disturbance plays a significant role in the progression and severity of diabetes and its complications, especially DKD [6–8]. Circulating amino acids are both biomarkers and causal agents of microvascular and mortality outcomes of diabetic patients [9, 10]. Amino acids contribute to augmented renal hemodynamic response and glomerular hyperfiltration in diabetic patients [11]. BCAAs are significantly associated with incident and following renal impairment of both type 1 and type 2 diabetes mellitus [12, 13].

In turn, the kidney directly impacts circulating metabolites through a series of mechanisms, like glomerular filtration, active tubular secretion, reabsorption, and passive reabsorption [14]. Being a crucial site for the metabolism and reabsorption of amino acid and glucose [11, 15, 16], the kidney also takes part in modulating glucose synthesized from amino acid, which is called gluconeogenesis. This organ also takes charge of synthesizing glycine, tyrosine, and arginine from 4-hydroxyproline, citrulline, and phenylalanine, respectively [17]. Approximately 30-80% of peripheral insulin is removed by the kidney as its important role in circular insulin clearance [18, 19]. Renal dysfunction changes the amino acid metabolism, which leads to inflammation, defects in immune response, insulin resistance, mitochondrial abnormalities, and protein energy wasting [18, 20, 21].

Our previous studies found that artemether exhibits renal protective effects in both T1DM and T2DM mice through regulating mitochondrial function [22, 23]. However, the effect of artemether on regulating renal metabolism, especially amino acid metabolism, has not been reported. Thus, this study is aimed at exploring the function of artemether in adjustment of renal amino acid metabolism in both T1DM and T2DM mice. Our findings may provide metabolic evidence of artemether in treating DKD.

## 2. Materials and Methods

### 2.1. Animals and Preparation of Diabetic Mouse Models

Male C57BL/6J mice were purchased from Guangdong Medical Laboratory Animal Center. Male db/db mice (BKS.Cg-Dock7^m+/+^Lepr^db^/Nju) and lean wild-type control mice were purchased from the Model Animal Center of Nanjing University. All mice were housed in the Center Animal Facility at Shenzhen Graduate School of Peking University. Mice were permitted a one-week acclimation to specified pathogen free laboratory conditions before the experiments start. All mice were housed under a 12 h/12 h light/dark cycle. Animal studies were performed in accordance with relevant guidelines and were approved by the Animal Ethics Committee of Guangzhou University of Chinese Medicine.

T1DM was established in 8-week-old C57BL/6J mice by intraperitoneal injection of STZ (Sigma-Aldrich, St. Louis, MO, USA) at 55 mg/kg/day (dissolved in citrate buffer) body weight for 5 consecutive days. Nondiabetic control (T1D-ctrl) mice received intraperitoneal injection of an equal volume of citrate buffer. Fasting blood glucose (FBG) of all mice was monitored 72 hours after the last injection. Animals with FBG concentrations ≥ 16.7 mmol/L were used as T1DM mice. Mice were randomly assigned into three groups: group 1—T1D-ctrl group, fed with a regular diet; group 2—STZ group, i.e., untreated T1DM mice, fed with a regular diet; and group 3—T1DM mice treated with artemether (STZ+Art group), fed with a regular diet supplemented with artemether at 1.6 g/kg. The treatment started one week after the last injection of STZ and lasted for 8 weeks.

The 8-week-old db/db T2DM mice were randomly assigned to three groups: group 1—lean wild-type T2D control (T2D-ctrl) group; group 2—db/db group; and group 3—db/db+artemether (db/db+Art) group. Mice in the T2D-ctrl group and db/db group were fed a regular diet. Mice in the db/db+Art group were fed a regular diet supplemented with artemether at 0.67 g/kg. The treatment lasted for 12 weeks.

Artemether was purchased from Chengdu ConBon Biotech Company (Chengdu, China).

### 2.2. Tissue Preparation

At the end of the experiment, mice were sacrificed, and blood samples were collected. Kidneys were removed immediately, snap-frozen in liquid nitrogen immediately, and stored at -80°C for further analysis.

### 2.3. Light Microscopy

Paraffin-embedded kidney sections (3 *μ*m) were stained with Periodic acid-Schiff (PAS) and scanned with a slide scanner (Motic Easyscan Digital Slide Scanner, Xiamen, China) to assess renal morphological alterations. For each section, 30-40 glomerular tuft areas (GTAs) and 50-80 proximal tubular areas were measured and analyzed, according to a method described before [24].

### 2.4. Biochemistry Analysis

Urine albumin excretion was detected by the enzyme-linked immunosorbent assay (Bethyl Laboratories, Montgomery, USA) according to the manufacturer's instructions. FBG was monitored biweekly after the treatment begins by using blood glucose meter (Roche, Basel, Switzerland). Glycated HbA1c (HbA1c) was measured using the HbA1c analyzer (Trinity Biotech Premier Hb9210 HbA1c, Ireland). The levels of total protein (TP) and albumin in serum, levels of glucose, and N-acetyl-*β*-glucosaminidase (NAG) in urine were measured with an automatic biochemical analyzer (Roche, Basel, Switzerland). Urine was collected by using metabolic cages (Tecniplast, Buguggiate, Italy).

### 2.5. Total Protein Concentration Analysis

Total protein of the kidney cortex was extracted with RIPA lysis buffer (Cell Signaling Technology, Massachusetts, USA) containing 1 mM PMSF (phenylmethylsulfonyl fluoride, Sigma-Aldrich, St. Louis, USA); an automatic homogenizer (Precellys Evolution homogenizer, Bertin Instruments, France) was applied for tissue homogenization. Total protein concentrations were detected by using the Quick Start™ Bradford Protein Assay kit (Bio-Rad, Hercules, USA) according to the manufacturer's instructions.

### 2.6. Metabolomic Profiling

#### 2.6.1. Chemicals and Reagents

Standards (99%, purity) of amino acid metabolites were purchased from Sigma-Aldrich (St. Louis, MO, USA), which were used for preparation of standard solutions. To perform metabolite concentration calculation, standard solutions were gradient diluted into ten levels, respectively (Table S2). The gradient diluted standard solutions were mixed and then dried by using a TurboVap® blowdown evaporator (Biotage Sweden AB, United Kingdom) for later use.

HPLC-grade acetonitrile, HPLC-grade methanol, and HPLC-grade formic acid were purchased from Merck (Merck KGaA, Darmstadt, Germany).

#### 2.6.2. Sample Preparation

Renal cortex tissues (~12 mg) were homogenized in extraction solvent (methanol: ultrapure water = 4 : 5 [*v*/*v*]), containing an isotope internal standard, L-tryptophan (indole-D5, 98%, Cambridge Isotope Laboratories, USA) at 2.5 *μ*g/mL, and incubated overnight (~12 hours) in -80°C for protein precipitation. The extraction solutions were then centrifuged for 20 minutes at 17800 × *g* at 4°C. The supernatant was transferred into fresh EP tubes, 10 *μ*L aliquots from each sample was collected and pooled as quality control samples, and the extracts were dried by using an evaporator. Before detection, dried samples and mixture of standard solutions were reconstituted in reconstitution solvent (methanol: ultrapure water = 1 : 1 [*v*/*v*]); then, the reconstituted solution was vortexed for 30 seconds and centrifuged for 10 minutes at 17800 × *g* at 4°C. The supernatant was transferred into a fresh 2 mL LC-MS glass vial for UPLC/Q/TRAP-MS analysis [25, 26].

#### 2.6.3. UPLC-MS/MS Conditions

All analyses were performed by ultra-high-performance liquid chromatography (SHIMADZU, Japan), coupled to the AB SCIEX Q-Trap 5500 triple quadrupole mass spectrometer (AB SCIEX, Toronto, Canada). Data acquisition and preliminary analysis were performed by SCIEX OS (version 1.6.1.29803).

Chromatographic separation was performed on the Luna Omega 1.6 *μ*m Polar C18 reversed-phase column (Phenomenex, California, USA) with column temperature kept at 40°C. Mobile phase A (0.1% formic acid in ultrapure water, *v*/*v*) and mobile phase B (100% acetonitrile) were delivered at 0.3 mL/min. Gradient elution was as follows: 2-60% B at 0-3.2 min, 60% maintained at 3.21-3.5 min, and 2% B at 3.51-5 min to equilibrate the column before a new injection. The injection volume for all samples was set at 2 *μ*L.

Analytes were detected under positive ion multiple reaction monitoring (MRM) mode. Turbo ion spray source was set at a source temperature of 500°C. Ion spray voltage was 5500 V, gas one and gas two had a flow of 50 psi, the curtain gas had a flow of 25 psi, the CAD gas setting was “medium,” and the declustering potential and collision energy was optimized one by one according to the metabolite. Q1/Q3 mass and MRM conditions for each metabolite are listed in Table S1. Quality control (QC) samples were detected every 8 tissue samples to evaluate the stability of the analysis system. CV range of QC samples about all metabolites was 3% to 19% in T1DM and 2% to 18% in T2DM.

### 2.7. Statistical Analysis

Data analysis was conducted using IBM SPSS Statistics (version 25.0.0.1). Data are expressed as mean ± standard deviation (SD). The significance of treatment effects was determined by one-way analysis of variance (ANOVA) test or nonparametric test (Kruskal-Wallis *H* test). The *P* values < 0.05 were considered statistically significant.

The targeted metabolic profiling data was firstly acquired by using SCIEX OS to perform concentration calculation. Metabolite peak extraction and process were performed with SCIEX OS. The peak area and standard concentration were used to construct individual metabolite calibration curves. Representative calibration curves are listed in Figure S1. We selected tryptophan indole-D5 as the isotope internal standard. The recovery rate of tryptophan indole-D5 was 105% in T1DM and 96% in T2DM. Endogenous metabolite concentrations of the tissue extracts were calculated according to the calibration curves with each metabolite peak area. The concentrations of metabolite in each group were firstly adjusted by total protein concentrations and then corrected by tryptophan indole-D5 concentrations. Detected concentration of metabolites and adjusted content by total protein as well as dual-corrected data of T1DM and T2DM are presented in Tables S3–S8. Fold change data of metabolites based on the control group were assessed with the dual-corrected data and are presented in scatter plot and bar graph. Detected concentrations were then imported to MetaboAnalyst 5.0 (https://www.metaboanalyst.ca/) for further analysis. Considering that the ranges of all metabolites were skewed, logarithmic transformation (base 10) and normalization by median were performed prior to analysis. An unsupervised method, principal component analysis (PCA), was applied to acquire an overview of the variation among groups. The orthogonal partial least squares discriminant analysis (OPLS-DA) was used as the classification method to simulate the discrimination by visualizing the score plots. To validate the OPLS-DA model, the permutation tests using 20 random permutations were performed. Spearman's correlation analysis was performed to determine the relationship between kidney weight and kidney tissue amino acid metabolites.

## 3. Results

### 3.1. Artemether Treatment Protected against Hyperglycemia in Diabetic Mice

**Figure 1:**
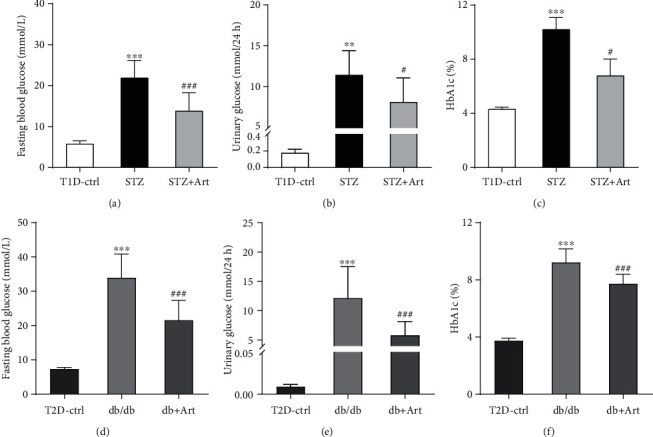
Artemether treatment protected against hyperglycemia in diabetic mice: (a, d) fasting blood glucose; (b, e) urinary glucose; (c, f) glycated HbA1c. For each group, *n* = 6-8. ^∗∗^*P* < 0.01 and ^∗∗∗^*P* < 0.001 vs. the Ctrl group. ^#^*P* < 0.05 and ^###^*P* < 0.001 vs. the STZ group or db/db group.

As shown in Figures 1(a) and 1(d), FBG of STZ mice and db/db mice were apparently higher than that of the T1D-ctrl and T2D-ctrl group, respectively, while after the artemether treatment, FBG significantly decreased. Urinary glucose was detected, as presented in Figures 1(b) and 1(e). Compared with their own control group, mice in the STZ and db/db group exhibited remarkable high levels of urinary glucose; artemether treatment effectively lowered the high levels. Generally, HbA1c can reflect the average blood glucose level in the past few weeks. In Figures 1(c) and 1(f), the application of artemether significantly decreased the increased levels of HbA1c in T1DM and T2DM mice.

Above all, indicated by significantly decreased FBG, urinary glucose, and HbA1c, artemether exerted positive efficacy in relieving hyperglycemia.

### 3.2. Artemether Treatment Mitigated Diabetic Kidney Hypertrophy and Normalized Kidney Size in T1DM and T2DM Mice

**Figure 2:**
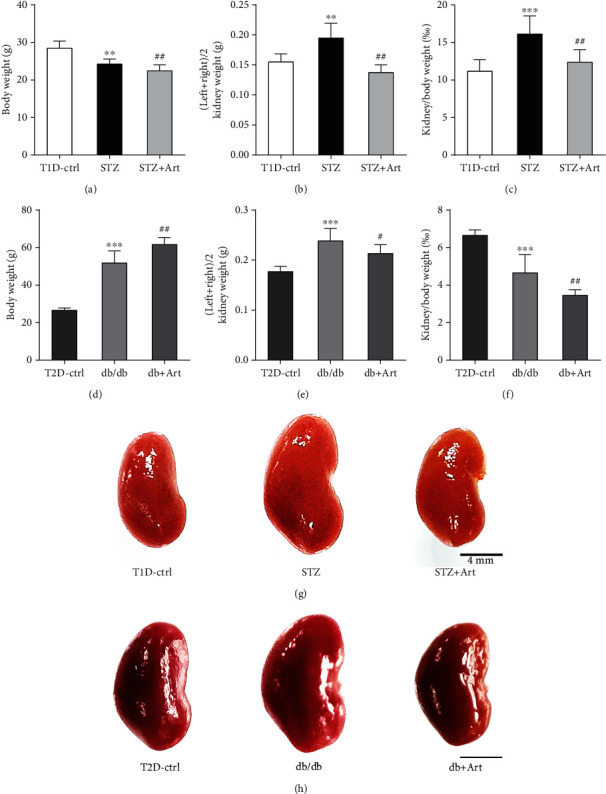
Artemether treatment mitigated diabetic kidney hypertrophy and normalized kidney size in T1DM and T2DM mice: (a, d) body weight of mice in each group; (b, e) kidney weight; (c, f) ratio of kidney weight/body weight; (g, h) macrograph of kidneys in each group. Scale bar: 4 mm. For each group, *n* = 6-8. ^∗∗^*P* < 0.01 and ^∗∗∗^*P* < 0.001 vs. the Ctrl group. ^#^*P* < 0.05 and ^##^*P* < 0.01 vs. the STZ group or db/db group.

Renal hypertrophy is a distinguished character of early DKD, which can be manifested as enlarged kidney size. As shown in Figures 2(b) and 2(e), kidney weight in the STZ group and db/db group was remarkably increased compared with that in the control group and apparently reduced after artemether treatment. As for T1DM, though average body weight of mice in the STZ group was a little bit heavier than that in the STZ+Art group (Figure 2(a)), the ratio of kidney weight to body weight was much higher in the STZ group (Figure 2(c)), which can be attributed to the remarkably increased kidney weight. Type 2 diabetic mice, including both the db/db group and db/db+Art group, showed significantly increased body weight as well as kidney weight compared with the control group (Figures 2(d) and 2(e)). Apparently, artemether played a role in reducing kidney weight and thus lowered the ratio of kidney weight to body weight (see Figure 2(f)).

As proven by significantly reduced kidney weight and the lowered ratio of kidney weight to body weight, artemether treatment exerted effects in normalizing kidney size and attenuating kidney hypertrophy.

### 3.3. Artemether Treatment Attenuates Renal Pathological Injuries in T1DM and T2DM Mice

**Figure 3:**
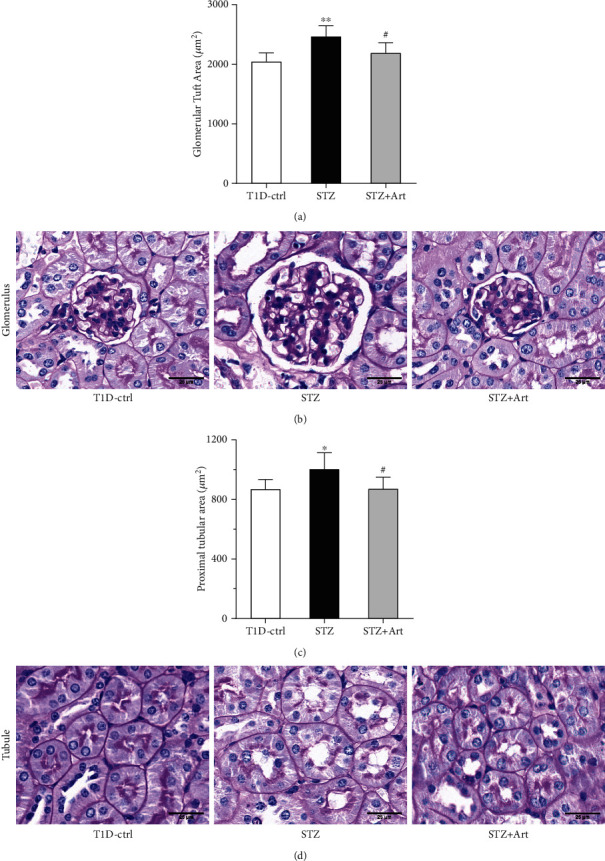
Artemether treatment attenuates renal pathological injuries in T1DM mice. (a) Bar graph indicating the glomerular tuft area of mice in each group. (b) Representative PAS staining images of glomerulus. Scale bar: 25 *μ*m. (c) Proximal tubular area of mice in each group. (d) Representative PAS staining images of the proximal tubule. Scale bar: 25 *μ*m. For each group, *n* = 6-8. ^∗^*P* < 0.05 and ^∗∗^*P* < 0.01 vs. the Ctrl group. ^#^*P* < 0.05 vs. the STZ group.

**Figure 4:**
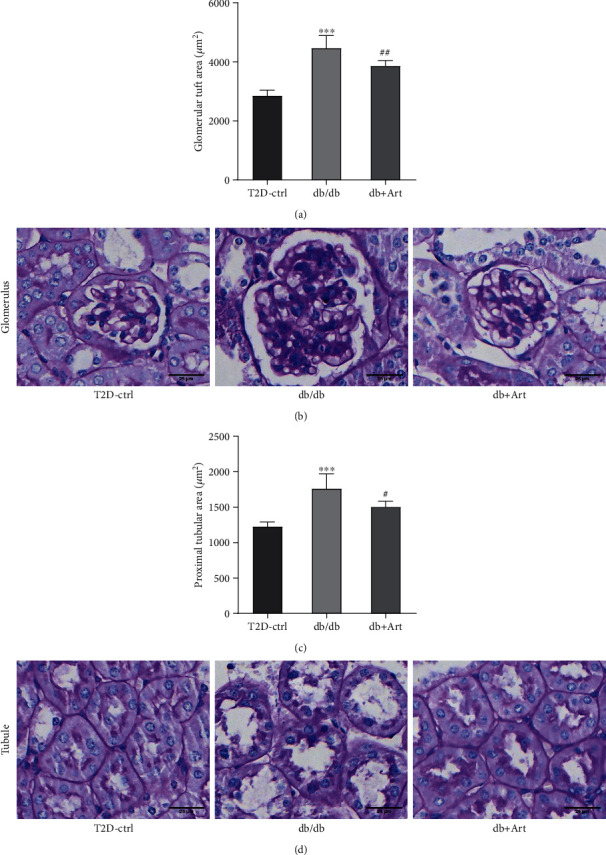
Artemether treatment attenuates renal pathological injuries in T2DM mice. (a) Bar graph indicating the glomerular tuft area of mice in each group. (b) Representative PAS staining images of glomerulus. Scale bar: 25 *μ*m. (c) Proximal tubular area of mice in each group. (d) Representative PAS staining images of the proximal tubule. Scale bar: 25 *μ*m. For each group, *n* = 6-8. ^∗∗∗^*P* < 0.001 vs. the Ctrl group. ^#^*P* < 0.05 and ^##^*P* < 0.01 vs. the db/db group.

It is acknowledged that the enlarged kidney in DKD results from hypertrophied glomeruli and dilated tubule. As shown in Figures 3(a) and 4(a), diabetic mice in T1DM and T2DM exhibited larger GTA, compared with control mice, respectively. Artemether treatment significantly attenuated the diabetic alterations in glomeruli (as shown in representative images in Figures 3(b) and 4(b)). Dilated renal tubule is another pathological characteristic of early DKD, especially the proximal tubules. Diabetic mice in the STZ group (Figure 3(c)) and db/db (Figure 4(c)) group displayed a bigger proximal tubular area. Artemether therapy significantly reduced the size of the proximal tubules, represented by images in Figures 3(d) and 4(d). The current results revealed that artemether treatment on renal pathological alterations was consistent with its effect on kidney weight.

### 3.4. Effect of Artemether on Urinary Albumin Excretion, Urinary NAG Levels, Serum Total Protein, and Serum Albumin Levels in T1DM and T2DM Mice

**Figure 5:**
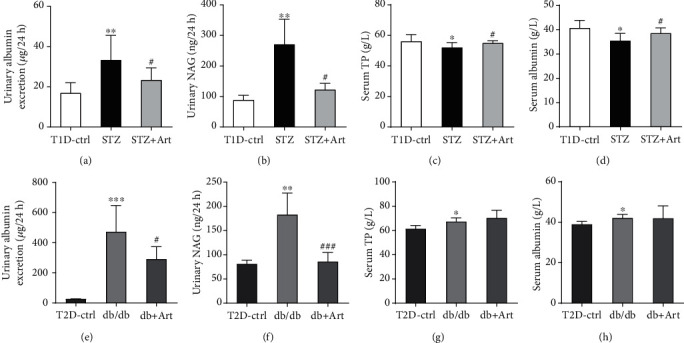
Effect of artemether on urinary albumin excretion, urinary NAG levels, serum total protein, and serum albumin levels in T1DM and T2DM mice: (a, e) 24-hour urinary albumin excretion; (b, f) urinary NAG levels; (c, g) serum total protein levels; (d, h) serum albumin levels. *n* = 6-8 each group. ^∗^*P* < 0.05, ^∗∗^*P* < 0.01, and ^∗∗∗^*P* < 0.001 vs. the Ctrl group. ^#^*P* < 0.05 and ^###^*P* < 0.001 vs. the STZ group or db/db group.

Albuminuria is another crucial characteristic of DKD. According to Figures 5(a) and 5(e), urinary albumin excretion maintained high levels in the STZ group and db/db group but was markedly reduced after the artemether treatment. As an important tubular injury marker, urinary NAG levels in both the STZ group and db/db group were obviously elevated (Figures 5(b) and 5(f)). Artemether significantly reduced the enhanced NAG levels in two models. We further measured the levels of total protein and albumin in serum; as shown in Figures 5(c) and 5(d), STZ mice exhibited much lower albumin and total protein levels in serum, and artemether treatment noticeably reversed it. Nevertheless, artemether did little effect on serum total protein and albumin levels in T2DM mice (see Figures 5(g) and 5(h)).

### 3.5. PCA and OPLS-DA of Amino Acid Metabolites in T1DM and T2DM Mice

**Figure 6:**
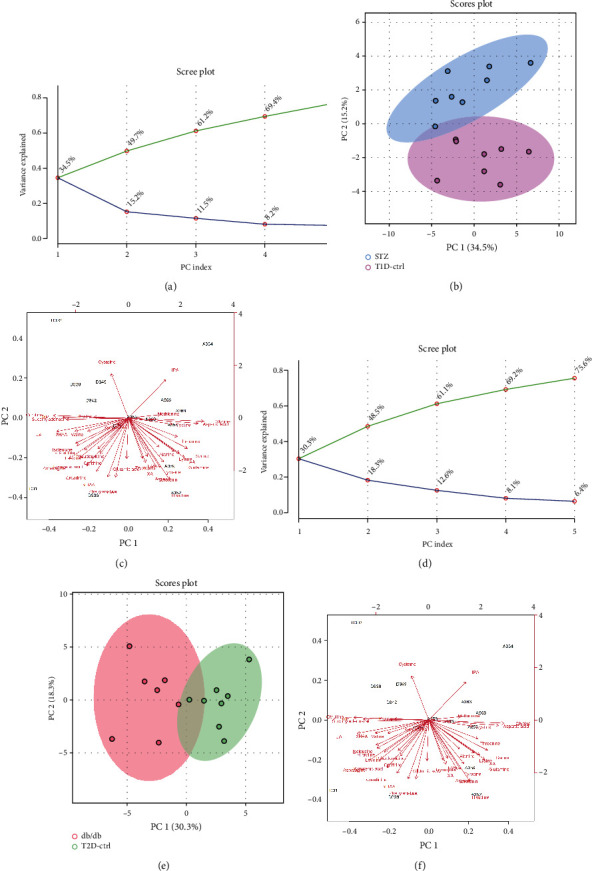
Principal component analysis of amino acid metabolites in T1DM and T2DM mice. Scree plots displayed top 5 PCs in T1DM (a) and T2DM (d) mice. The green line on top showed the accumulated variance explained; the blue line underneath showed the variance explained by individual PC. 2D PCA score plot in T1DM (b) and T2DM (e) mice. Biplot of amino acid metabolites in T1DM (c) and T2DM (f) mice. Images were presented by STZ or db/db group vs. corresponding control group.

We analyzed metabolites associated with amino acid metabolism in the kidney cortex of both T1DM and T2DM mice to investigate metabolic changes in DKD. As shown in Figure 6, PCA was performed to investigate intrinsic diabetic model-related clusters within the datasets and to identify outliers; samples from the STZ group and db/db group were only partially separated from the corresponding control group.

**Figure 7:**
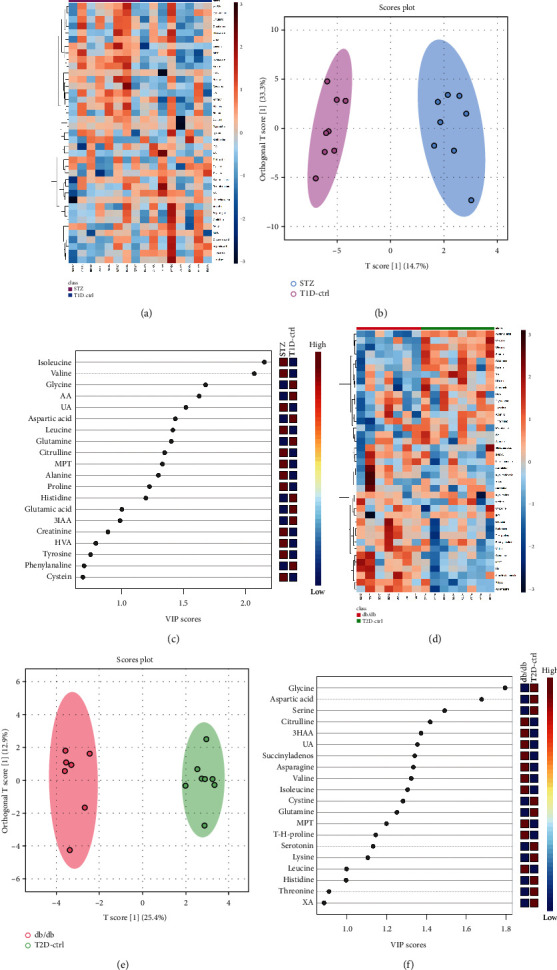
Heatmaps and OPLS-DA of amino acid metabolites in T1DM and T2DM mice. Hierarchical clustering heatmaps of metabolites in T1DM (a) and T2DM (d) mice. (b) OPLS-DA score plot of T1D-ctrl and STZ group (validated by permutation tests, *R*^2^*Y* = 0.97, *Q*^2^ = 0.771). (e) OPLS-DA score plot of T2D-ctrl and db/db group (validated by permutation tests, *R*^2^*Y* = 0.982, *Q*^2^ = 0.882). Importance scores of metabolites in T1DM (c) and T2DM (f) mice, respectively. The colored boxes on the right indicated the relative concentrations of the corresponding metabolite in each group under study.

A heatmap provides intuitive visualization of the data. Each colored cell on the map corresponds to a concentration value in the data, with samples in rows and metabolites in columns. As shown in Figures 7(a) and 7(d), according to the concentration value, samples from each group were matched with amino acid metabolites and thus presented in different colors for high values in red tones and low values in blue tones. Then, the OPLS-DA was used to improve separation between groups. The OPLS-DA score map screened the selection of different metabolites and was used to distinguish the separation between groups after PCA. It indicated that there was a significant separation between the two groups in type 1 and type 2 diabetic mice, respectively (see Figures 7(b) and 7(e)). Variable importance in projection (VIP) represents the ability to extract variables of differentiation between groups, and variables with VIP values greater than 1 (VIP > 1) were listed (Figures 7(c) and 7(f)). Permutation tests were performed to validate OPLS-DA models, and the results confirmed its validity. In comparison with their control groups, similar alterations of amino acids can be observed in both T1DM and T2DM mice, which indicated that the two models of DKD did share similarities in metabolic features.

### 3.6. Effects of Artemether on the Levels of Amino Acid Metabolites in the Diabetic Kidney Cortex

**Figure 8:**
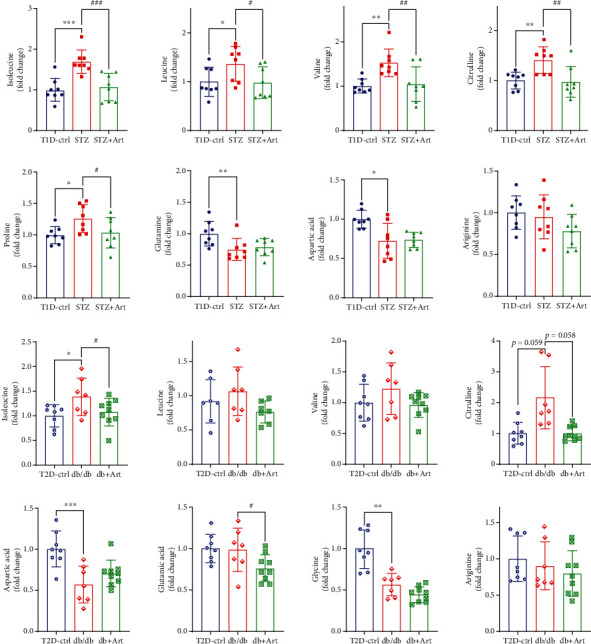
Significant differential amino acids in the kidney cortex of T1DM and T2DM mice. Detected amino acid concentration was dual corrected by total protein and tryptophan indole D5. Metabolite contents exhibiting significant differences and/or sharing consistent tendency with or without artemether treatment were presented in scatter plots with bar. *n* = 6-8 each group. ^∗^*P* < 0.05, ^∗∗^*P* < 0.01, and ^∗∗∗^*P* < 0.001 vs. the Ctrl group. ^#^*P* < 0.05, ^##^*P* < 0.01 and ^###^*P* < 0.001 vs. the STZ or db/db group.

Amino acid metabolites exhibiting significant differences and/or sharing consistent tendency in both T1DM and T2DM mice were selected and are presented in Figure 8 and Tables S7–S8. Levels of leucine, isoleucine, valine, citrulline, and proline were increased in both 2 types of diabetic mice, whereas they were decreased after the application of artemether. Several metabolites were downregulated in both 2 types of diabetic mice, including glutamine, glutamic acid, aspartic acid, arginine glycine, lysine, histidine, and methionine. In the presence of artemether, glutamine and aspartic acid were upregulated.

Though fundamentally distinct in pathogenesis, pancreatic *β*-cell dysfunction is a key process in the development of both T1DM and T2DM. As for the amino acid metabolism of DKD, parities and disparities still coexist in T1DM and T2DM. Here, we focused on the similarities, trying to figure out their role in DKD. Although some of the changes were of no statistical significance, most metabolites shared consistent tendency when compared with their own control group, in either T1DM or T2DM mice, as well as after treatment with artemether. It is believed that artemether regulated renal cortex amino acid metabolism in both T1DM and T2DM mice and therefore relieved diabetic renal injuries.

### 3.7. Effects of Artemether on the Ratio of Amino Acid Metabolites in the Diabetic Kidney Cortex

**Figure 9:**
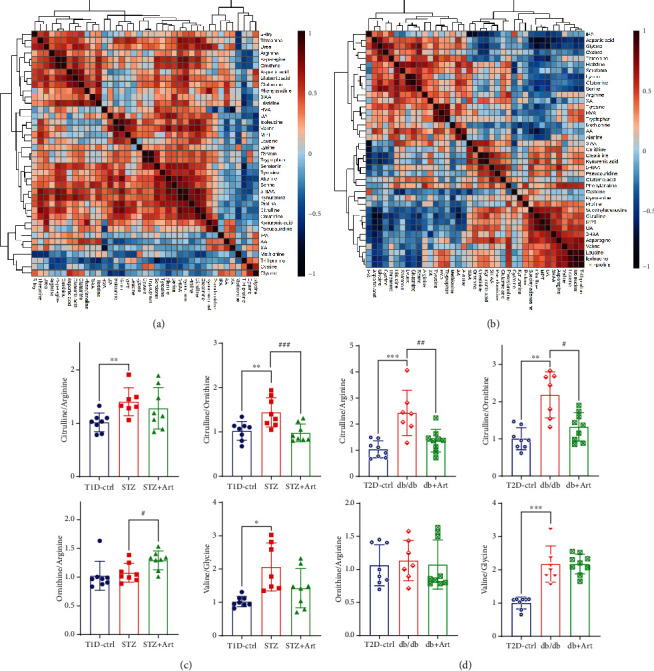
Correlation heatmaps and ratio of metabolites in the kidney cortex. Correlation heatmaps of T1DM (a) and T2DM (b) show correlations between metabolites. Scatter plots with bar are the ratio of correlated metabolites in T1DM (c) and T2DM (d). *n* = 6-8 each group. ^∗^*P* < 0.05, ^∗∗^*P* < 0.01, and ^∗∗∗^*P* < 0.001 vs. the Ctrl group. ^#^*P* < 0.05, ^##^*P* < 0.01, and ^###^*P* < 0.001 vs. the STZ or db/db group.

Persistent conversions from one amino acid to another are deeply rooted in the metabolism process of amino acids which involved complicate intraorgan and interorgan coordination. Here, we listed the expression ratios of correlated metabolites, trying to elucidate the possible connections and dispositions. According to Figure 9, the ratio of renal citrulline to arginine was increased in both types of diabetic mice group, and artemether treatment could lower this increased ratio. Ratio of citrulline to ornithine was obviously enhanced in both types of diabetic mouse group, which meant a reduced production in ornithine and urea, and application of artemether decreased the upregulated levels. The results indicated that the conversion from citrulline to arginine and ornithine might be somehow impeded or inhibited, which suggested that diabetic renal injuries could lead to imbalance between ureagenesis and NO production. The application of artemether could adjust citrulline-arginine metabolism, restore the balance of the urea cycle and NO cycle, and therefore relieve DKD injuries.

### 3.8. Correlation Analysis between Expression of Amino Acid Metabolites and Kidney Weight

**Figure 10:**
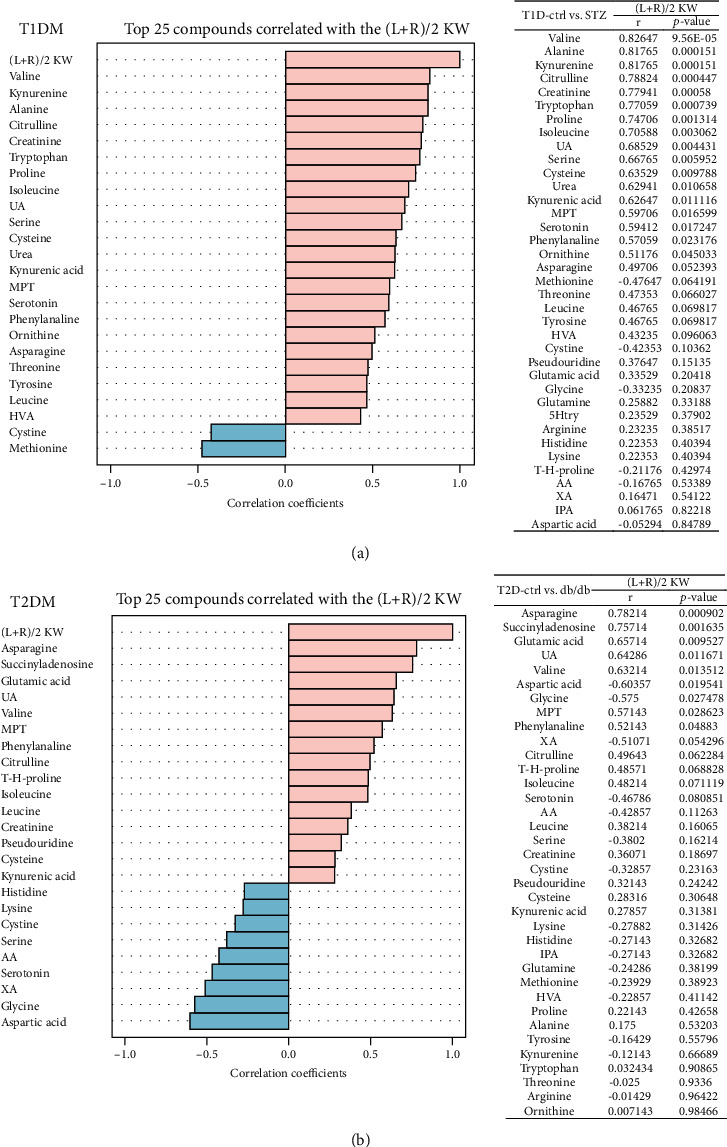
Correlation analysis between expression of amino acid metabolites and kidney weight. Correlation analysis between expression of metabolites and kidney weight in T1DM (a) and T2DM (b) mice. Tables on the right side listed the corresponding correlation coefficients and *P* values of metabolites. (L+R)/2 KW: (left+right)/2 kidney weight.

Renal enlargement is crucial in the pathogenesis of DKD. Correlation analysis was performed to find out the potential link between kidney weight and amino acid metabolites. As shown in Figure 10(a) and its upper right table, there were nearly 20 amino acid metabolites positively correlated with STZ mice's kidney weight, which meant that the increased expression of these metabolites may account for the increment in kidney mass. As shown in Figure 10(b), in T2DM mice, there were 7 significantly correlated amino acid metabolites with either positive or negative relationship. Among those correlated metabolites, attention should be paid to citrulline, valine, leucine, and isoleucine.

## 4. Discussion

This study demonstrates that artemether could protect against hyperglycemia and albuminuria and thus mitigate renal hypertrophy in both type 1 and type 2 diabetic mice. We firstly reported that the positive effects of artemether exerted on DKD mice could be attributed to the adjustment of amino acid metabolism. Artemether treatment could prominently reverse the enhanced renal cortex citrulline in mice. In T1DM mice, artemether could downregulate the enhanced branched-chain amino acid—valine, leucine, and isoleucine. In both type 1 and type 2 models, most amino acids could be improved by artemether with a consistent tendency.

As to the clinical phenotype, this study got similar results with our previous studies [22, 23]. Mice treated with artemether got decreased fasting blood glucose, HbA1c, and urinary glucose levels. Urinary albumin excretion, urinary NAG, and serum albumin and total protein were ameliorated by artemether treatment. Renal hypertrophy evidenced by the ratio of kidney weight to body weight was also relieved by artemether application.

**Figure 11:**
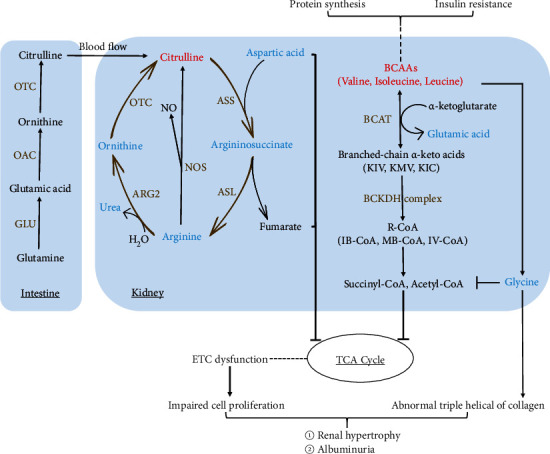
Schematic depicting changes of amino acid metabolism, urea cycle, and their connection in the renal cortex after artemether treatment. Metabolites lowered by artemether treatment are shown in blue whereas metabolites increased by artemether treatment are shown in red. Characters in brown are key enzymes involved. Lines with arrows in brown made the urea cycle. GLU: glutaminase; OTC: ornithine transcarbamylase; OAC: ornithine aminotransferase; ASS: argininosuccinate synthase; ASL: argininosuccinate lyase; BCAT: branched-chain aminotransferase; BCKDH: branched-chain ketoacid dehydrogenase; NOS: nitric oxide synthase; ETC: electron transport chain; ARG2: arginase 2.

In agreement with previous studies [6, 27–29], our results found an increased citrulline level in kidney cortex tissue of two diabetic animal models. Citrulline, a nonproteinogenic amino acid, is the substrate of catabolic as well as the product of anabolic processes and reported to be an important factor in stimulating protein synthesis [30]. At the whole-body level, citrulline is almost exclusively synthesized from glutamine and proline in the enterocytes. Citrulline is catalyzed into argininosuccinate by ASS (argininosuccinate synthase) and converted to arginine by ASL (argininosuccinate lyase) [31]. Both enzymes ASS and ASL are localized in the proximal tubule of the kidney. The kidney is a major site of arginine synthesis, and about 85% of the gut-derived citrulline is taken up for converting into quantitative arginine [32]. The rate-limiting step of the conversion process includes the following: (1) the delivery and uptake rate of citrulline to the renal cortex [33], and (2) aspartic acid, the substrate of argininosuccinate, also a potential metabolic node modulated by glucose. In our study, the enhanced renal cortex citrulline level in type 1 diabetic mice and high correlation coefficient (Figure 10, *r* = 0.78824, *P* = 0.00044745) between citrulline and kidney weight indicated that upregulated citrulline coincided with the diabetic hyperfiltration and hypertrophy states. The upregulated renal plasma flow in the diabetic hyperfiltration stage resulted in increased delivery of citrulline to the kidney, while the declined expressions of aspartic acid and arginine indicated that the citrulline-arginine conversion process was limited. The citrulline-arginine pathway adjusts the balance between arginase and NOS and facilitates anabolic or proinflammatory/apoptotic processes [34]. The ratio of citrulline to ornithine was obviously enhanced in T1DM and T2DM mice, with a meaning of decreased ornithine production and also ureagenesis, during the process. This result showed an imbalance between ureagenesis and NO production in diabetic renal injury. Artemether could lower the increased renal citrulline, reverse the ratios of citrulline to arginine and citrulline to ornithine, and enhance the level of aspartic acid in both models. The results gave us the evidence that artemether could regulate citrulline-arginine metabolism, restore the balance of the urea cycle and NO cycle, and further exhibit antihypertrophy function. Aspartic acid could be generated from glutamine decarboxylation during electron transport chain (ETC) inhibition [35]. The same changing tendency of glutamine and aspartic acid as well as their high correlation coefficient (*r* = 0.59341 in T1DM and *r* = 0.45155 in T2DM) in our study agreed with their findings (see Figure 11).

In line with other studies [12, 36, 37], elevated leucine, isoleucine, and valine, also known as BCAAs, and reduced glycine were observed in both renal cortexes. Artemether treatment could improve the BCAAs and glycine disorder. Elevated BCAAs and low glycine are reported to be associated with insulin resistance [10]. Branched-chain aminotransferase (BCAT) could catalyze BCAAs with *α*-ketoglutarate and produce branched-chain keto acids as well as glutamate. In mitochondria, leucine, valine, and isoleucine are, respectively, catalyzed into acetyl-CoA, propionyl-CoA, and *α*-methylbutyryl-CoA, with the enzyme, branched-chain keto acid dehydrogenase (BCKDH). Acetyl-CoA, propionyl-CoA, and 2-methylbutyryl-CoA all enter the tricarboxylic acid (Krebs) cycle and lead to ATP formation [38, 39]. BCAA oxidation could conduce to acylation of mitochondrial enzymes [40]. Glycine plays vital roles in organic metabolism and acts as a precursor of serine, purines, creatine, sarcosine, glutathione, and collagen. As required in stabilizing the triple helix of collagen, glycine has a critical role in maintaining collagen structure [41]. Glycine could limit the acyl-CoA excretion in the form of acyl-glycine in urine [37]. Enhanced BCAAs could activate glycine deleting to dispose the excess NH_3_ generated by BCAAs and maintain nitrogen balance [42]. BCAA-restricted diet could increase tissue and plasma glycine level in a fatty rat model and ameliorate insulin resistance [37].

The previous study showed that artemether could promote mitochondrial function, adjust mitochondrial redox balance, and protect diabetic kidney. Our renal cortex amino acid results in diabetic mice agreed with these findings. Some intermediates of BCAA oxidation are found to be beneficial to acylation of mitochondrial enzymes [40]. Aspartic acid synthesis plays an important role in cell proliferation upon ETC inhibition [35]. ASS downregulation is another mechanism to support cell proliferation and suggests a metabolic link between pyrimidine synthesis and urea cycle [43]. Low intracellular arginine was reported to be associated with a shift from oxidative phosphorylation to glycolysis [44]. Arginine and aspartic acid are indispensable for appropriate mitochondrial respiration and genome integrity. Arginine starvation could lead to epigenetic silencing of mitochondrial ETC complex genes through histone acetylation [45]. Downregulated urea cycle products could shunt metabolites toward pyrimidine biosynthesis and away from arginine synthesis to support cell proliferation [46] (see Figure 11).

In conclusion, our data support that artemether could improve citrulline, BCAA catabolism, and urea cycle, reverse declined arginine and aspartic acid, modulate renal amino acid metabolism, facilitate nitrogen balance, inhibit cell proliferation and hypertrophy, ameliorate albuminuria, and resist diabetic renal injury.

## Data Availability

Data of this study are available from the corresponding authors on reasonable request.

## Abbreviations

T1DM: Type 1 diabetes mellitus
T2DM: Type 2 diabetes mellitus
DKD: Diabetic kidney disease
CKD: Chronic kidney disease
STZ: Streptozotocin
ART: Artemether
BCAAs: Branched-chain amino acids
HVA: Homovanillic acid
UA: Uric acid
HK: Hydroxykynurenine
XA: Xanthurenic acid
5HIAA: 5-Hydroxy-indole-3-acetic acid
T-H-proline: Trans-4-hydroxy-L-proline
3HAA: 3-Hydroxyanthranilic
MPT: 2-*α*-D-Mannopyranosyl-L-tryptophan
5Htry: 5-Hydroxy-L-tryptophan
3IAA: Indole-3-acetic acid
AA: Anthranilic acid
IPA: Indole-3-propionic acid
KYA: Kynurenic acid
UPLC-MS: Ultra-high-performance liquid chromatography-mass spectrometry
PCA: Principal component analysis
OPLS-DA: Orthogonal partial least square discriminant analysis
VIP: Variable importance in projection
GLU: Glutaminase
OAC: Ornithine aminotransferase
OTC: Ornithine transcarbamylase
ASS: Argininosuccinate synthase
ASL: Argininosuccinate lyase
ARG2: Arginase 2
BCAT: Branched-chain aminotransferase
BCKDH: Branched-chain ketoacid dehydrogenase
NOS: Nitric oxide synthase
ETC: Electron transport chain.

## Supplementary Materials

Supplementary Materials Figure S1: calibration curves of selected metabolites. Table S1: Q1/Q3 mass and MRM conditions for the selected metabolites. Table S2: gradient diluted and corresponding concentrations of standards. Table S3: detected concentrations of metabolites in T1DM mice. Table S4: detected concentrations of metabolites in T2DM mice. Table S5: metabolite content adjusted by total protein in T1DM mice. Table S6: metabolite content adjusted by total protein in T2DM mice. Table S7: metabolites corrected by total protein and tryptophan indole D5 in T1DM mice. Table S8: metabolites corrected by total protein and tryptophan indole D5 in T2DM mice.
