# Supplementary material for: Artemether Alleviates Diabetic Kidney Disease by Modulating Amino Acid Metabolism
Source: Biomed Res Int. 2022 May 11;2022:7339611. doi: 10.1155/2022/7339611 (PMC9117059; doi:10.1155/2022/7339611)
Supplement: Supplementary Materials — Figure S1: calibration curves of selected metabolites. Table S1: Q1/Q3 mass and MRM conditions for the selected metabolites. Table S2: gradient diluted and corresponding concentrations of standards. Table S3: detected concentrations of metabolites in T1DM mice. Table S4: detected concentrations of metabolites in T2DM mice. Table S5: metabolite content adjusted by total protein in T1DM mice. Table S6: metabolite content adjusted by total protein in T2DM mice. Table S7: metabolites corrected by total protein and tryptophan indole D5 in T1DM mice. Table S8: metabolites corrected by total protein and tryptophan indole D5 in T2DM mice. [file 7339611.f1.docx]

**Figure S1**

**Calibration curves of selected metabolites.**


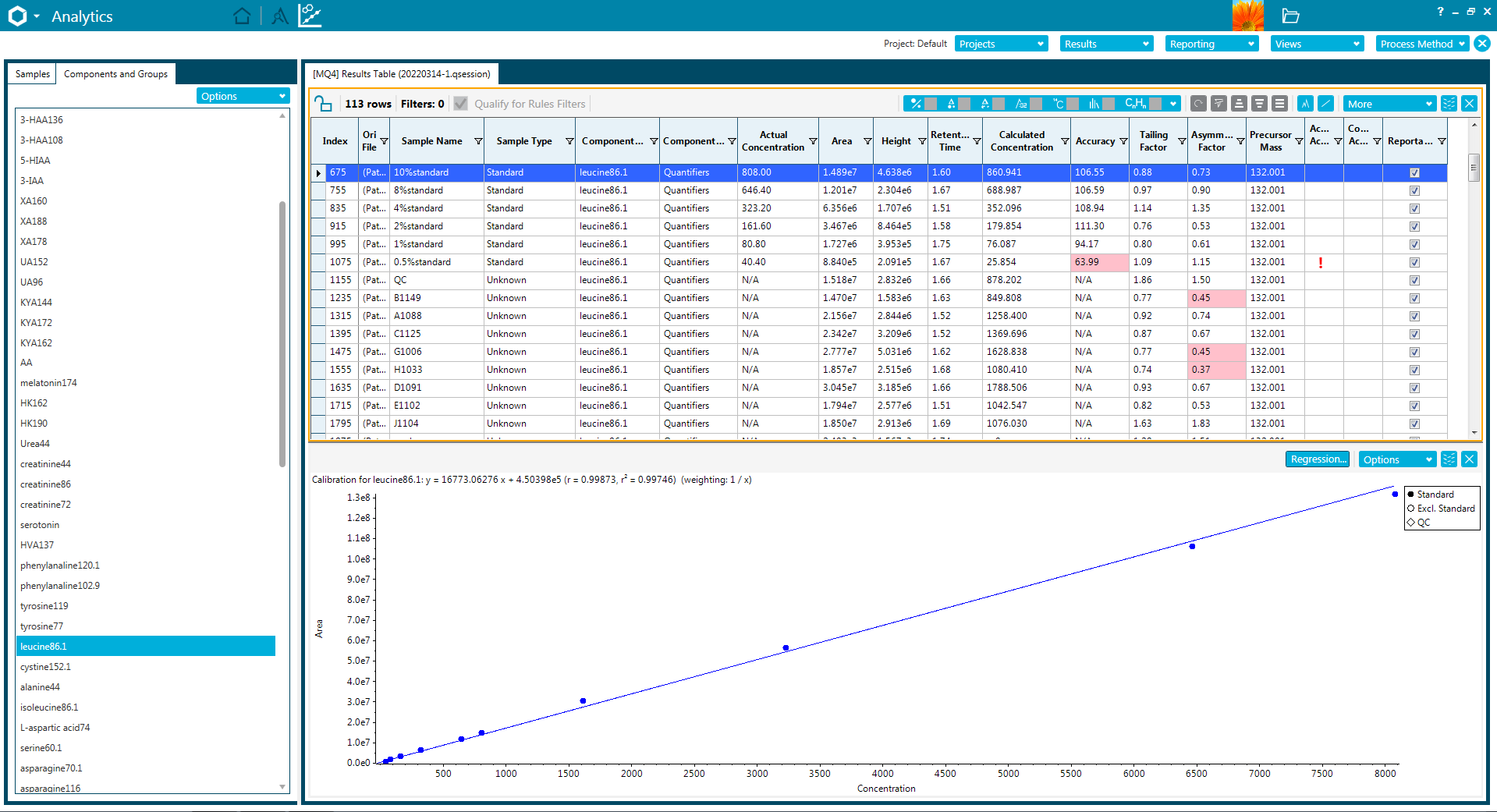


Leucine R^2^=0.99746


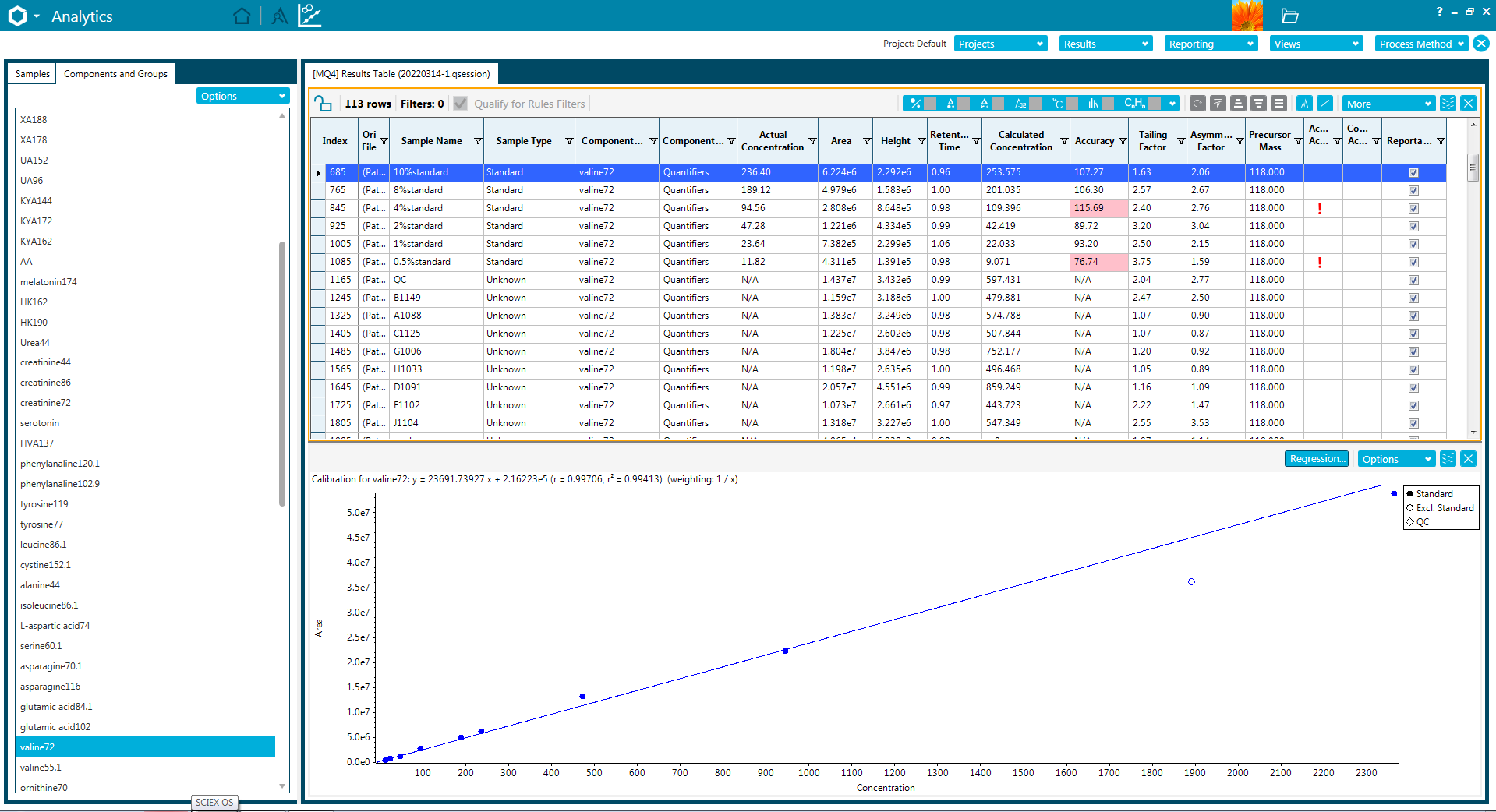


Valine R^2^=0.99413


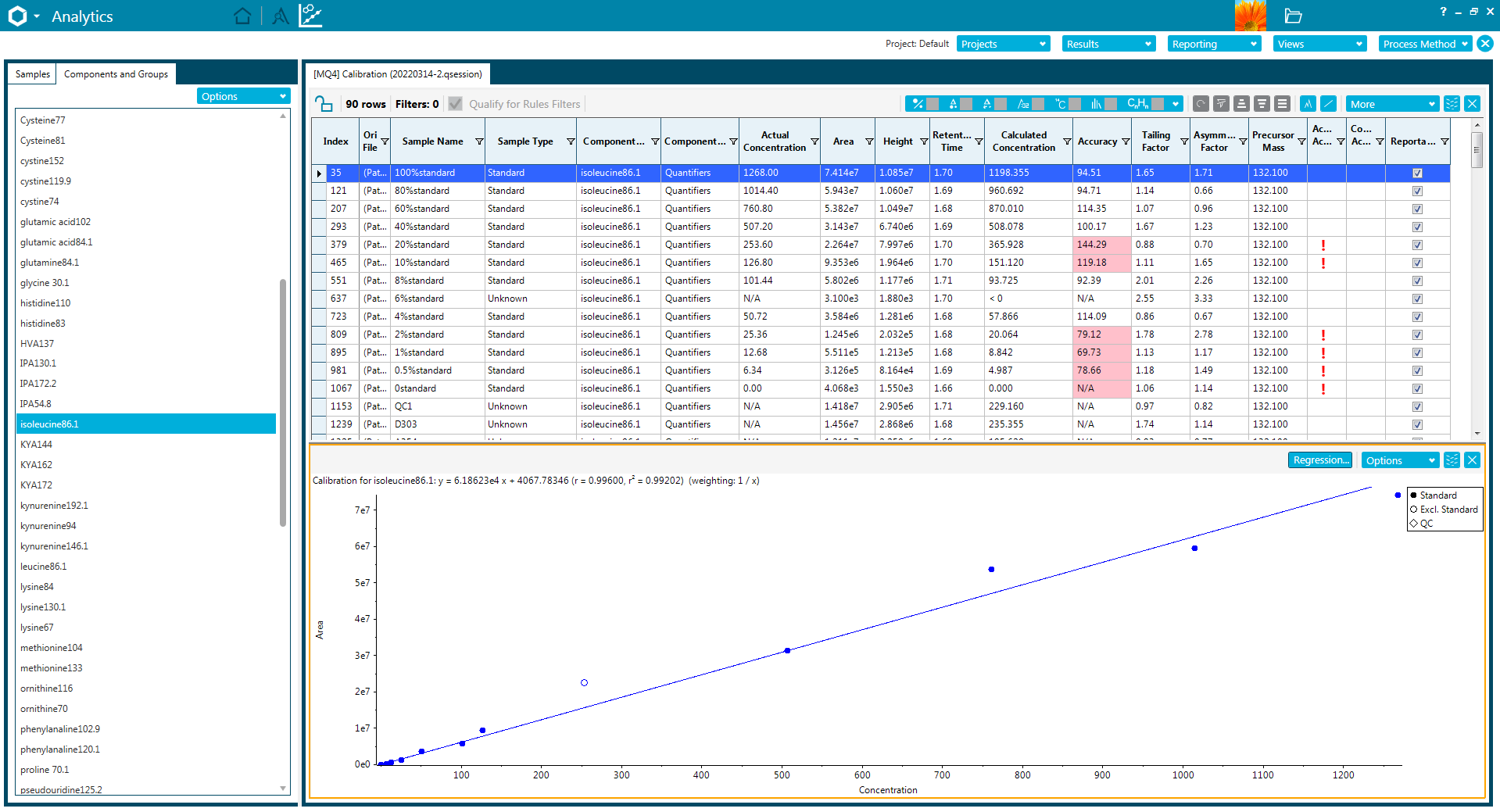


Isoleucine R^2^=0.99202


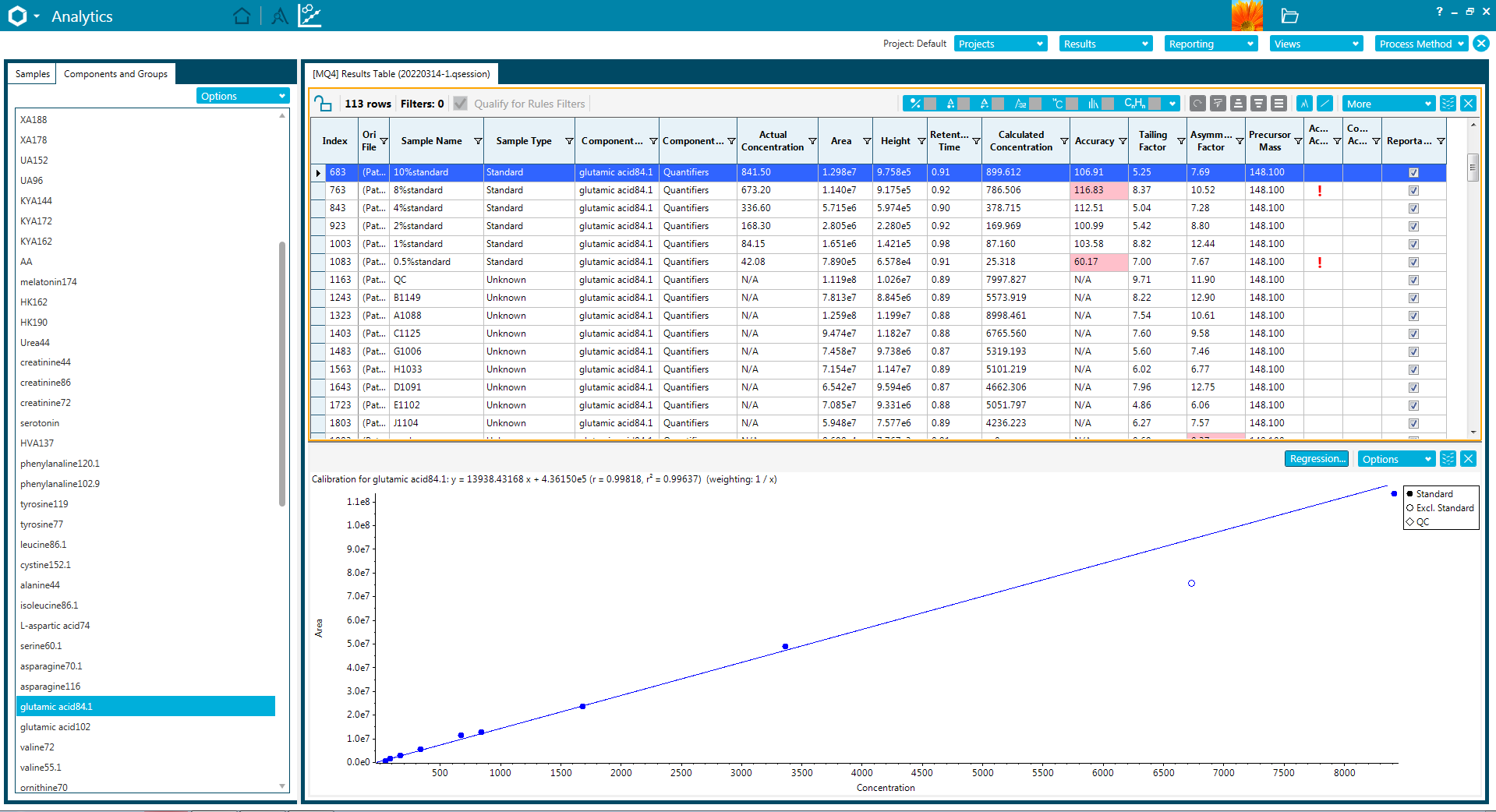


Glutamic acid R^2^=0.99637


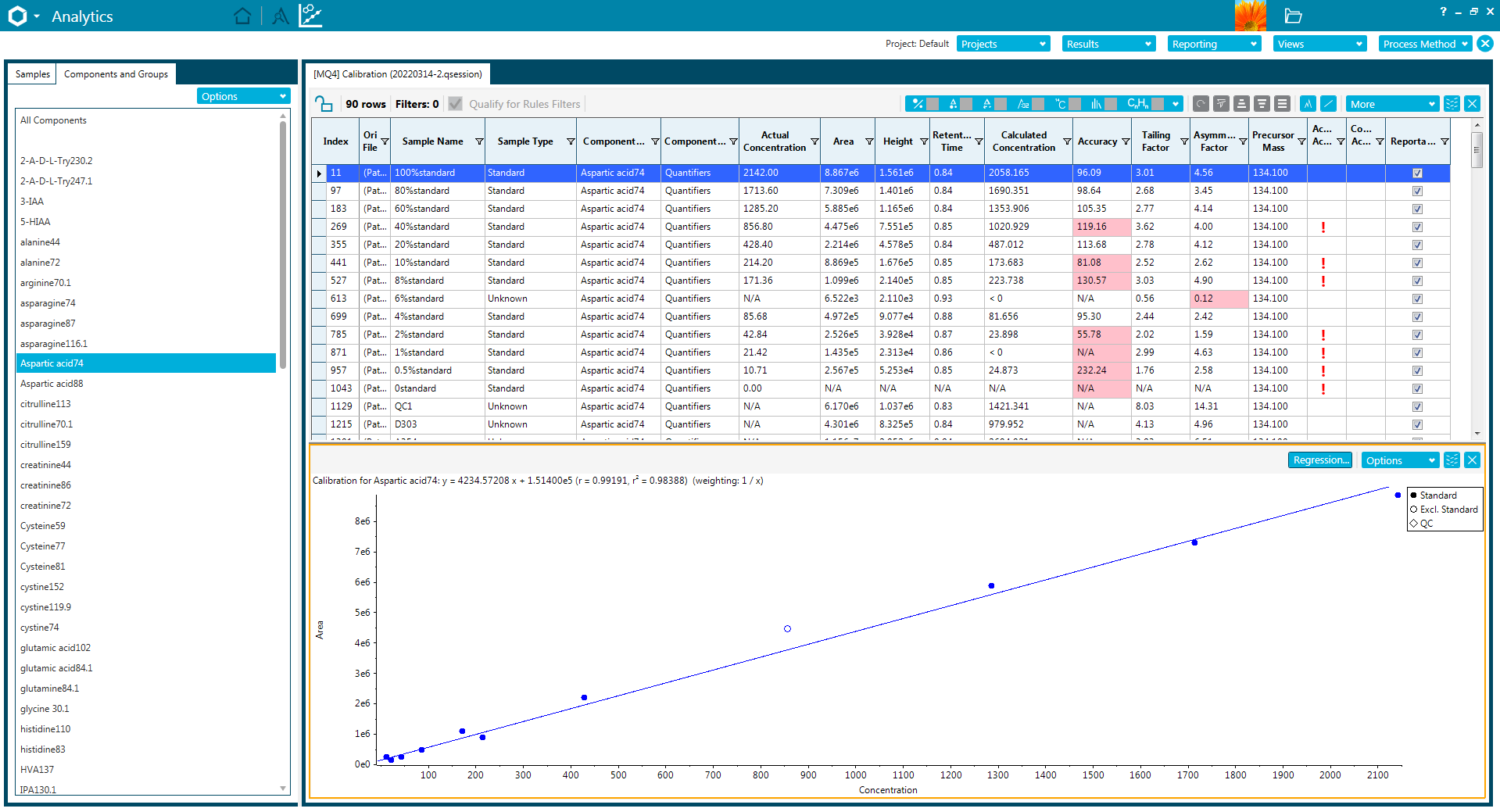


Aspartic acid R^2^=0.98388


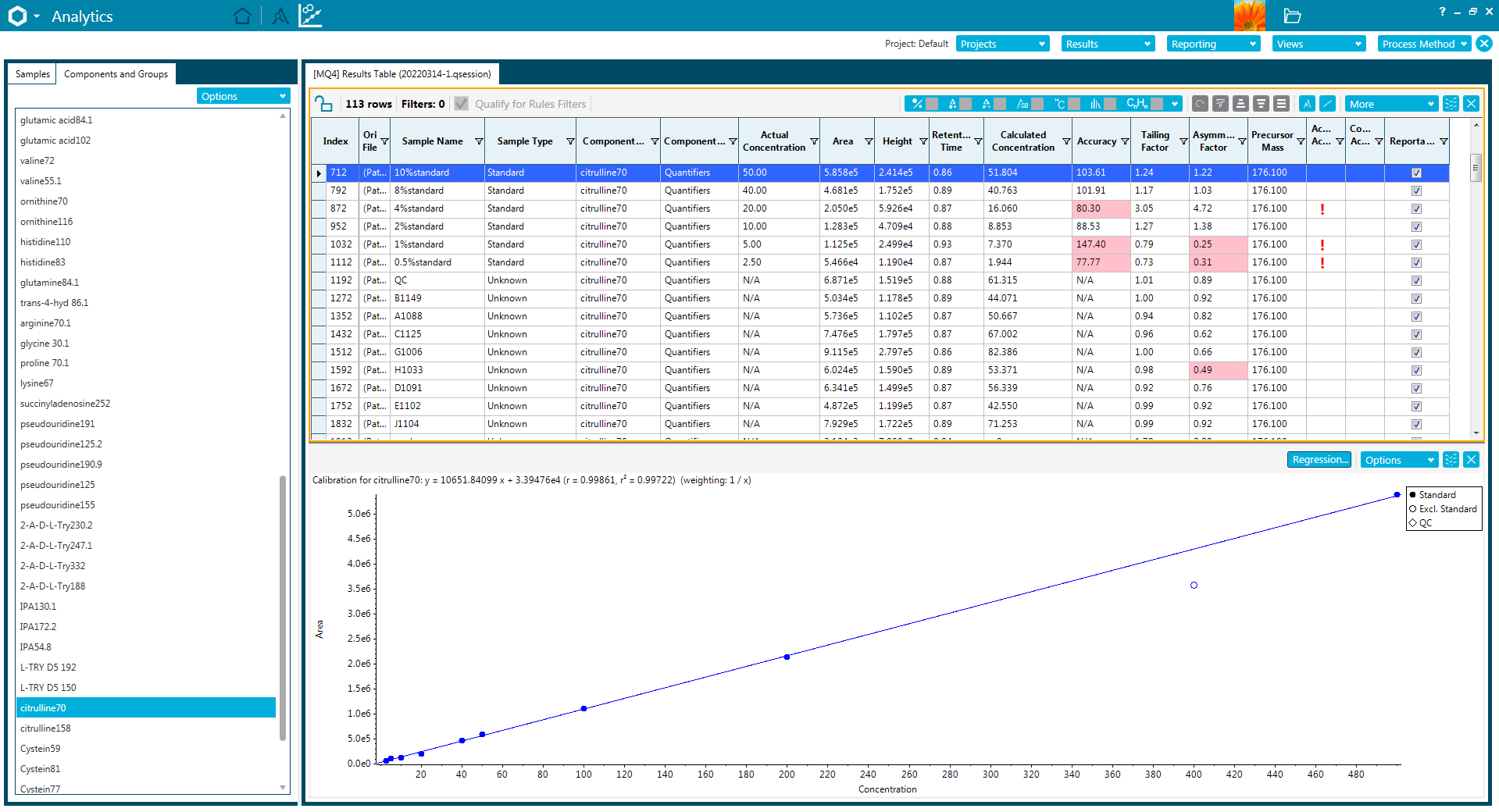


Citrulline R^2^=0.99722


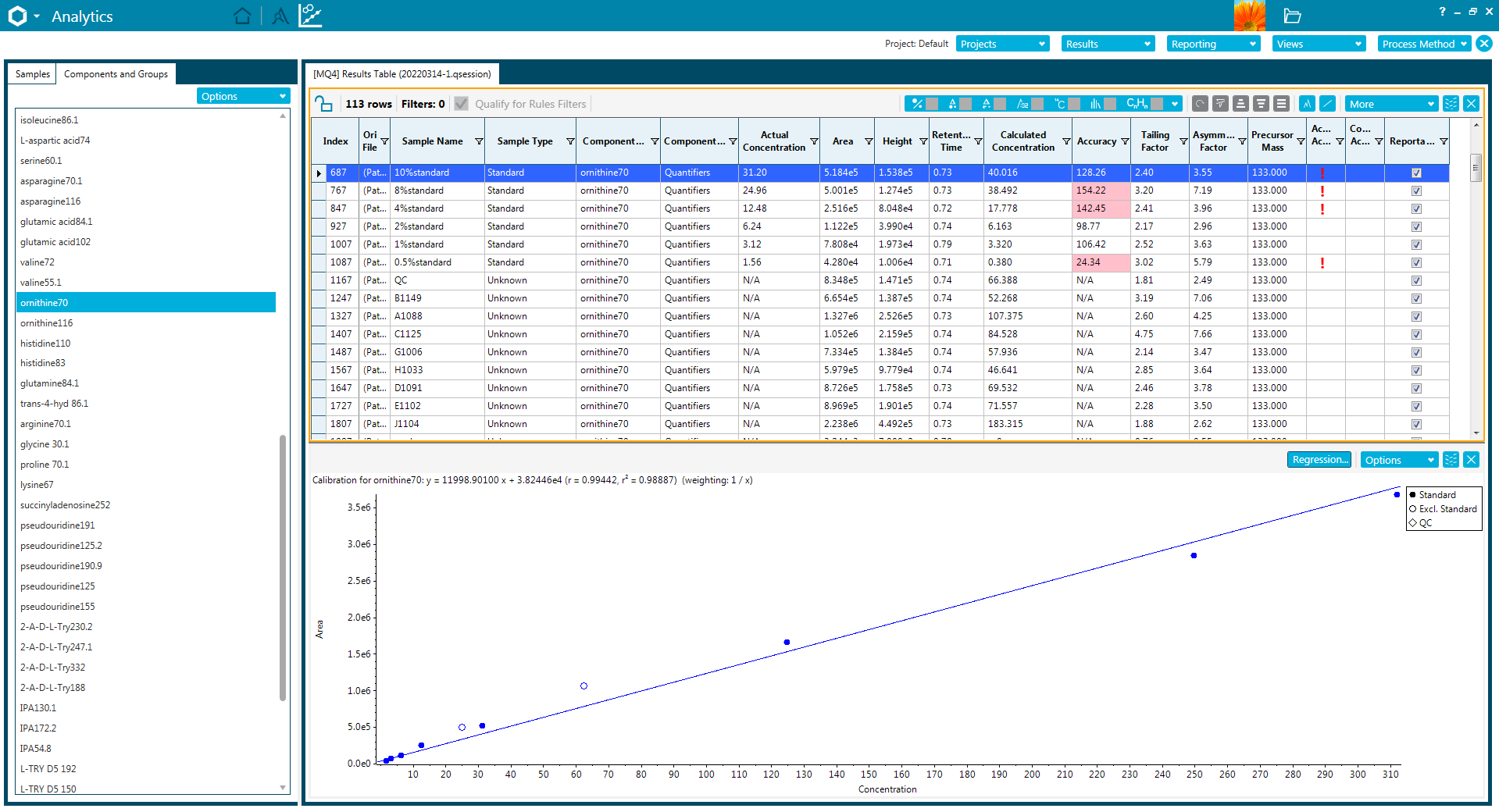


Ornithine R^2^=0.98887


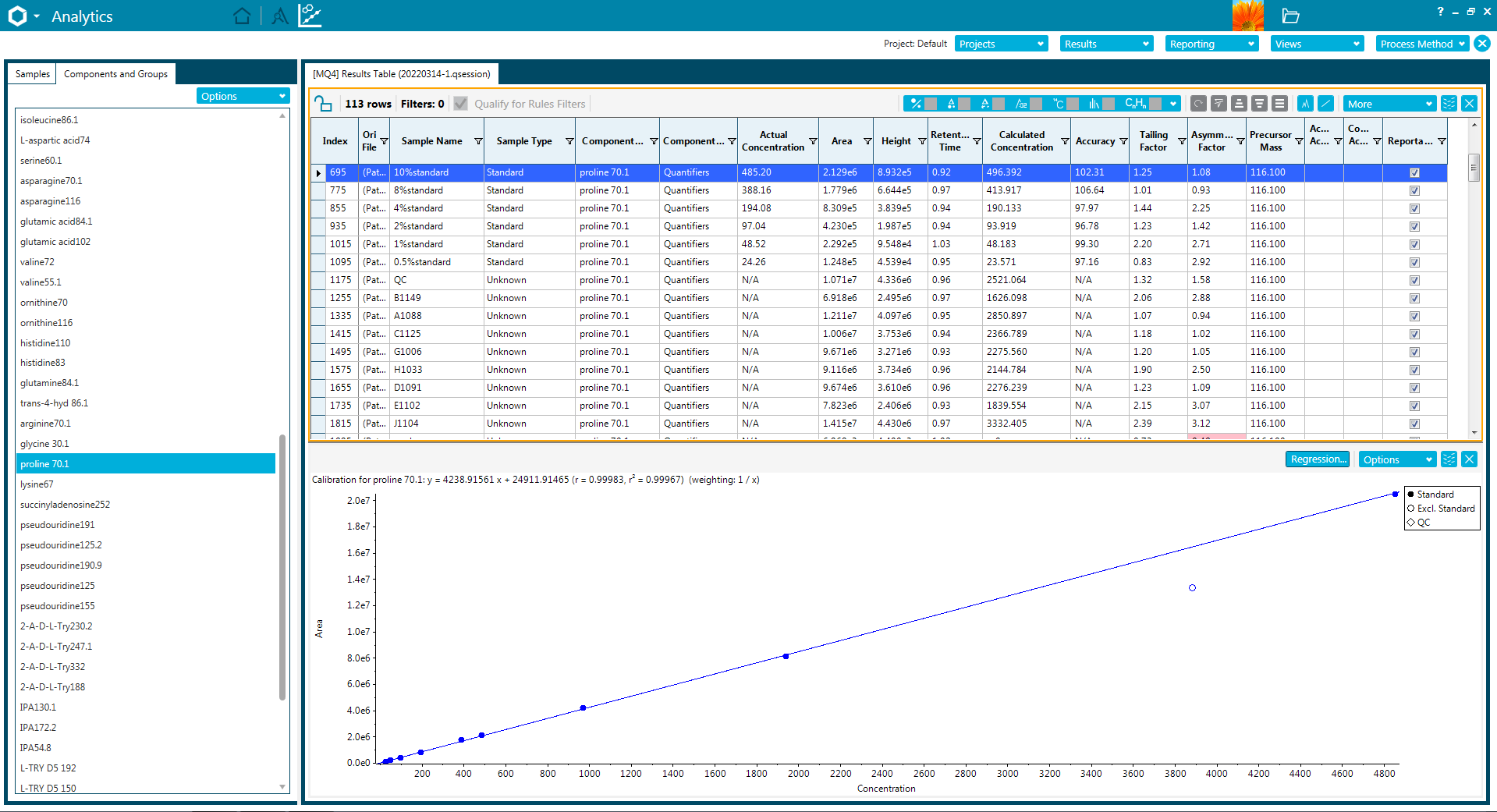


Proline R^2^=0.99967


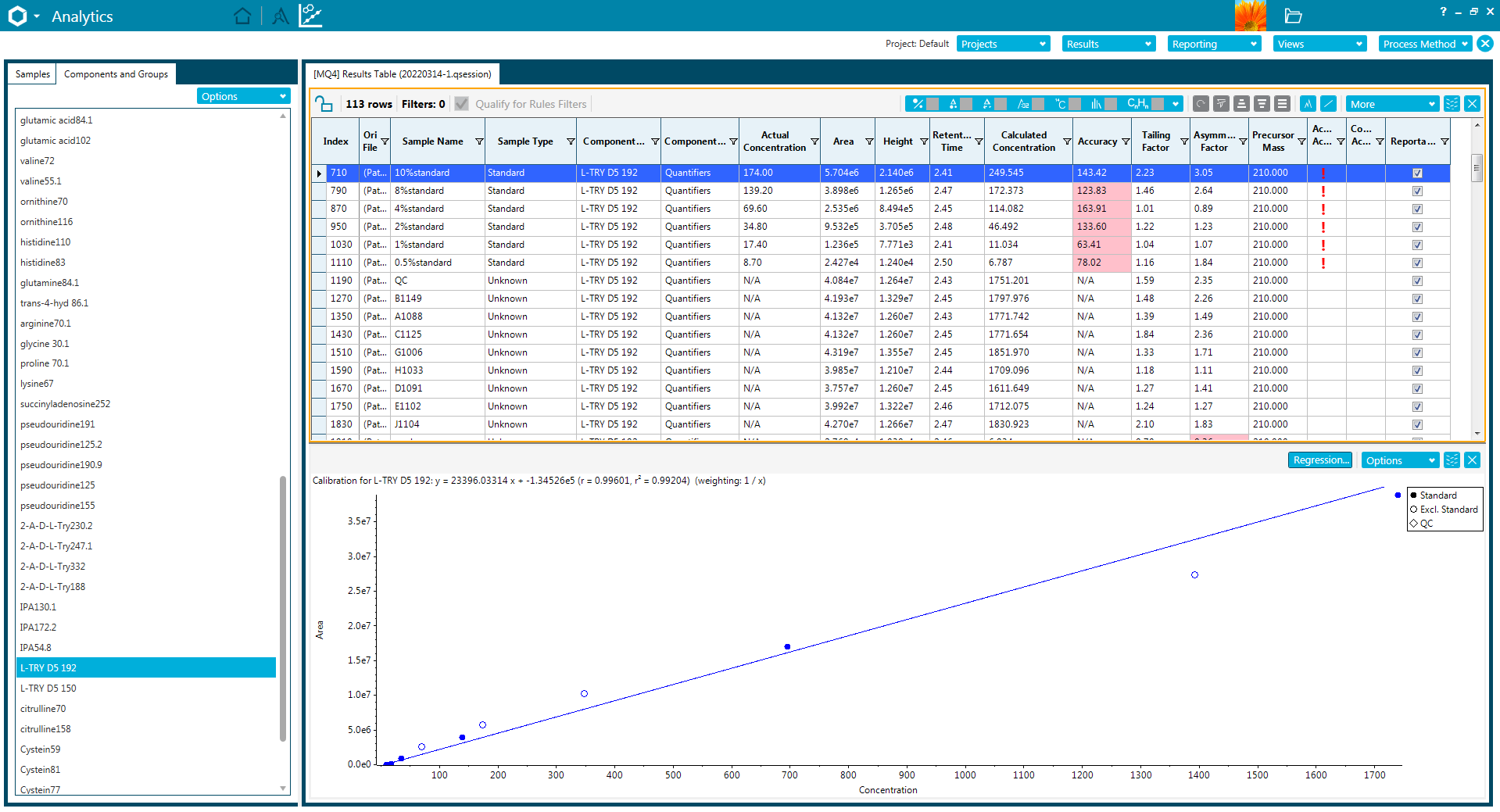


L-Tryptophan (INDOLE-D5) R^2^=0.99204


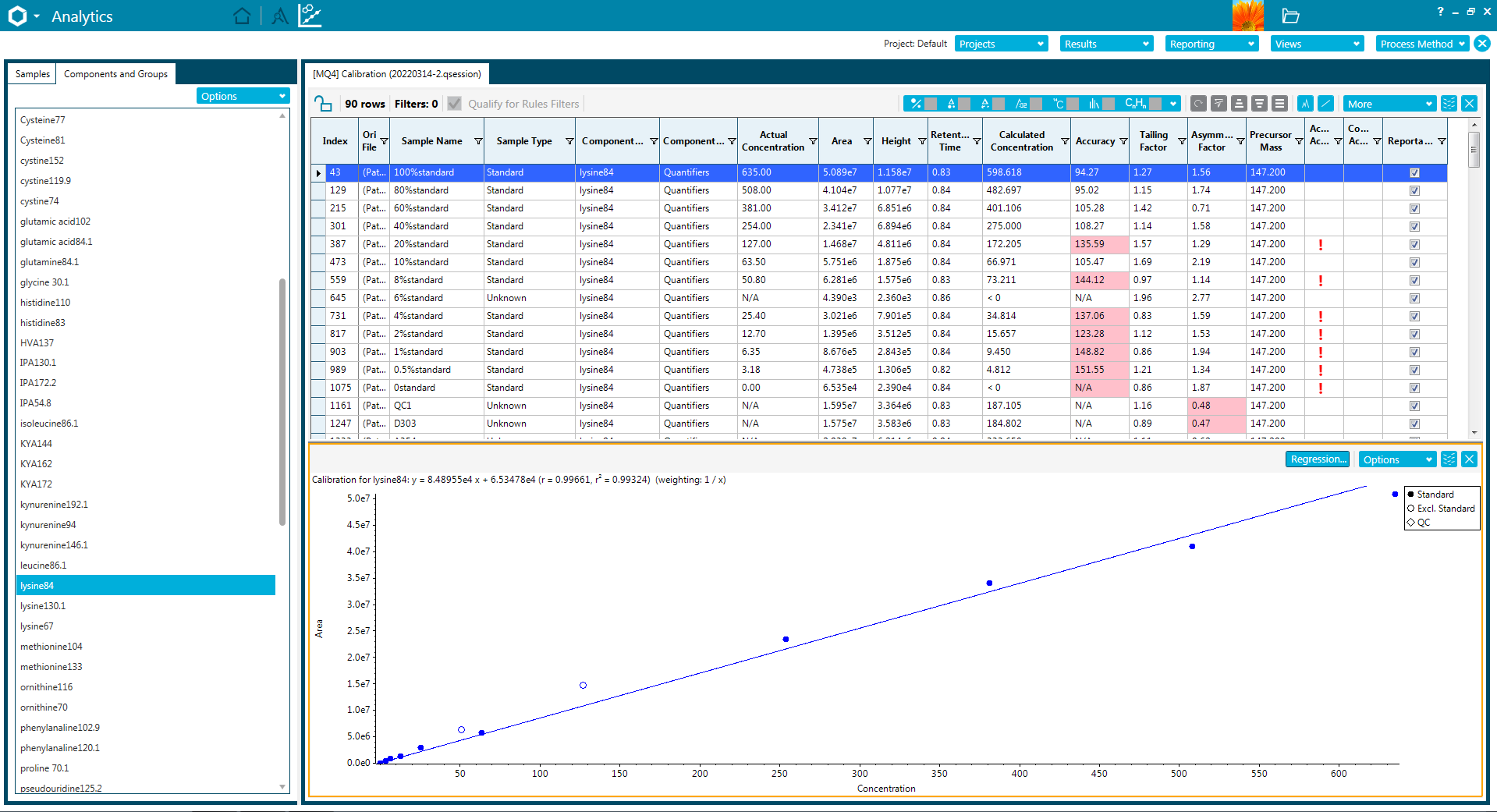


Lysine R^2^=0.99324


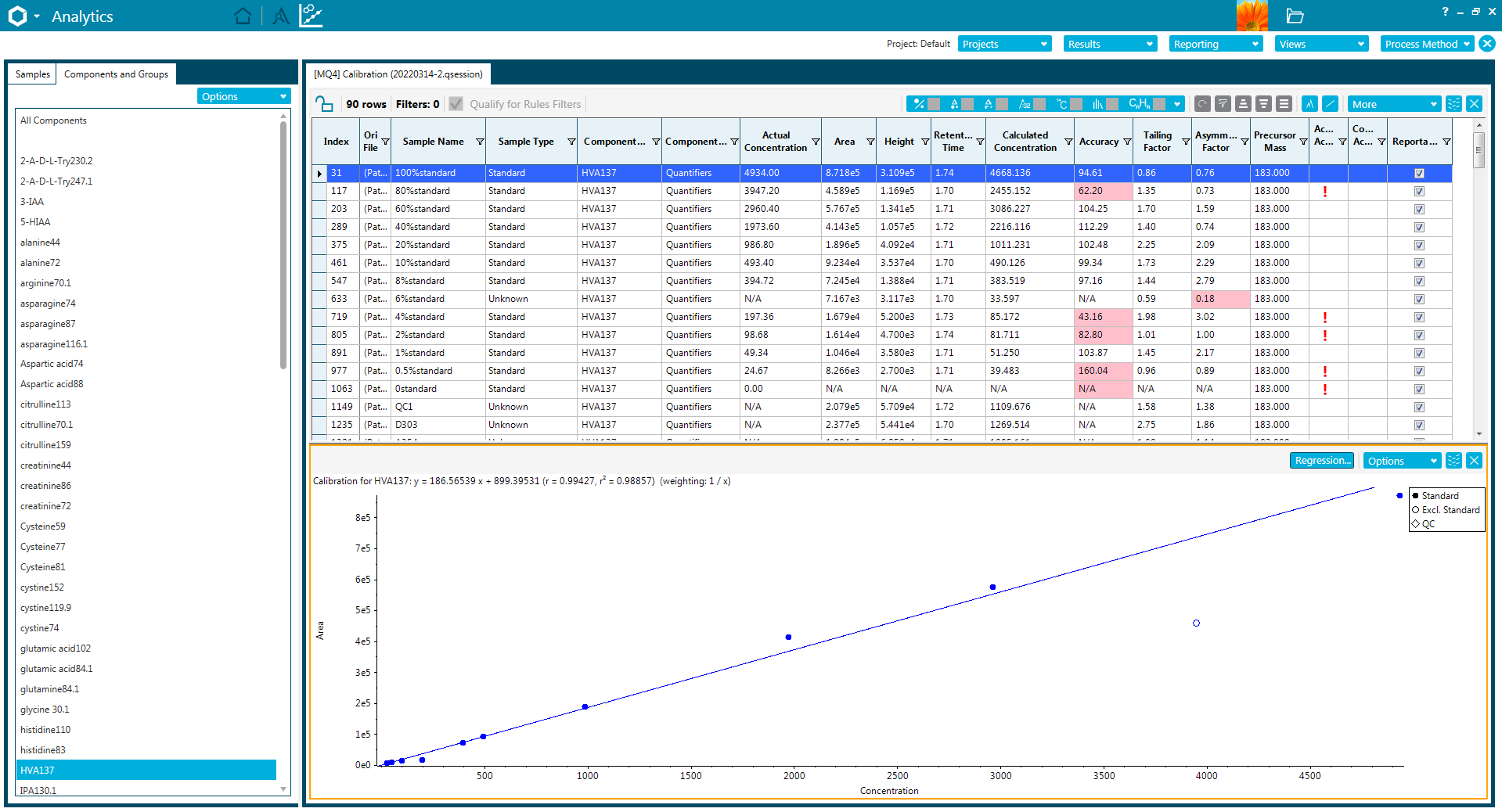


Homovanillic acid R^2^=0.9885


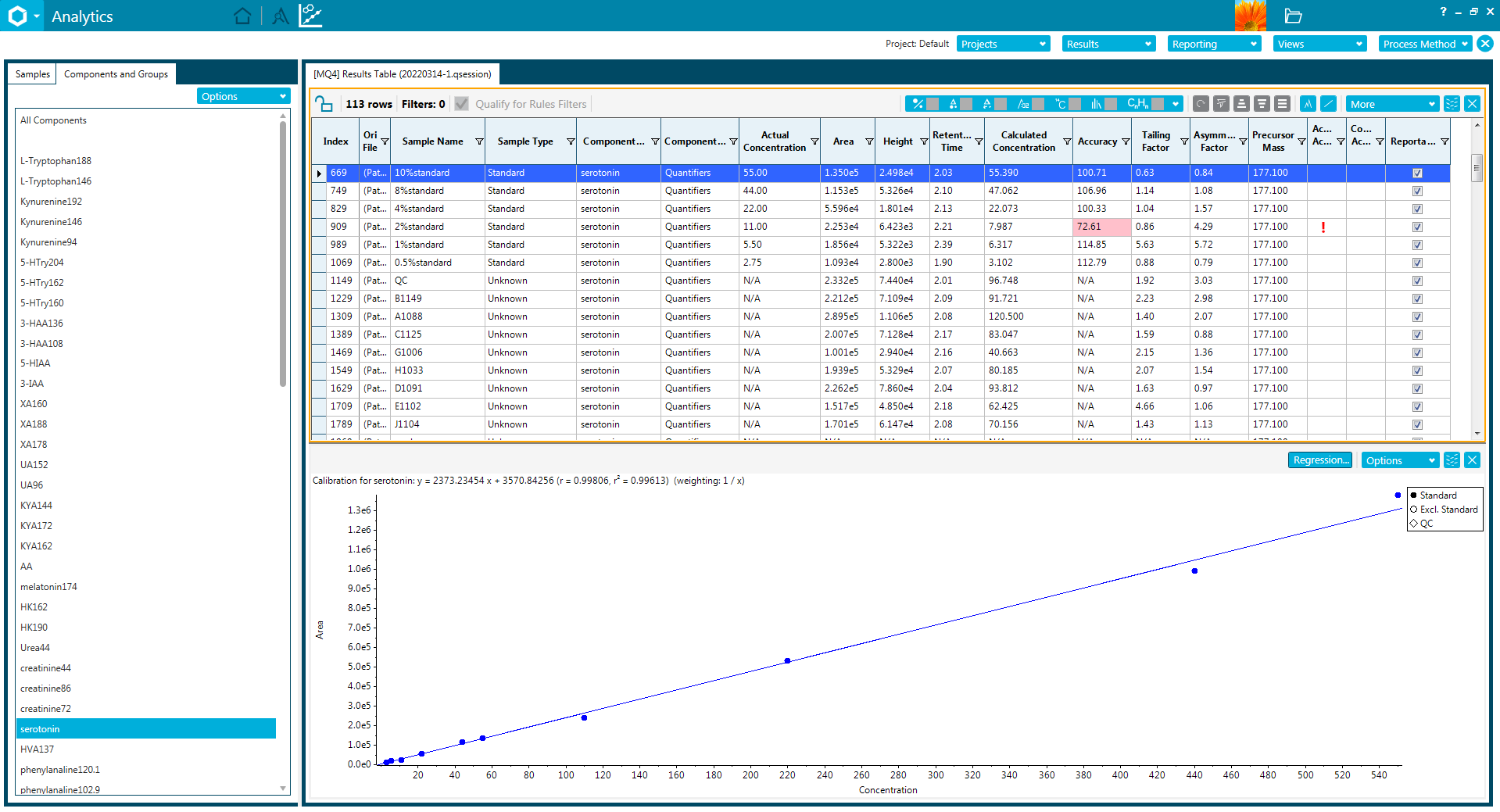


Serotonin R^2^=0.99613


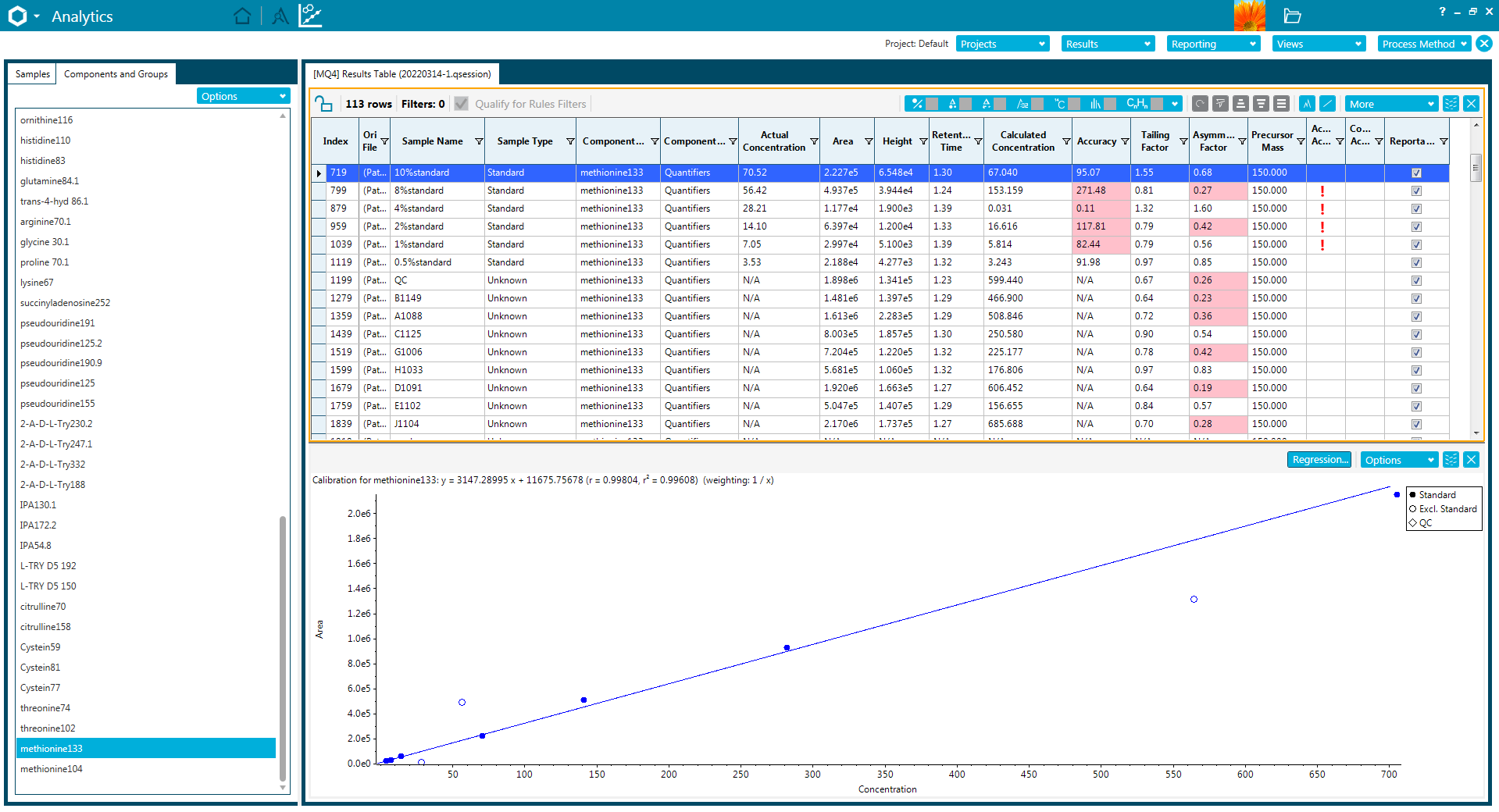


Methionine R^2^=0.99608


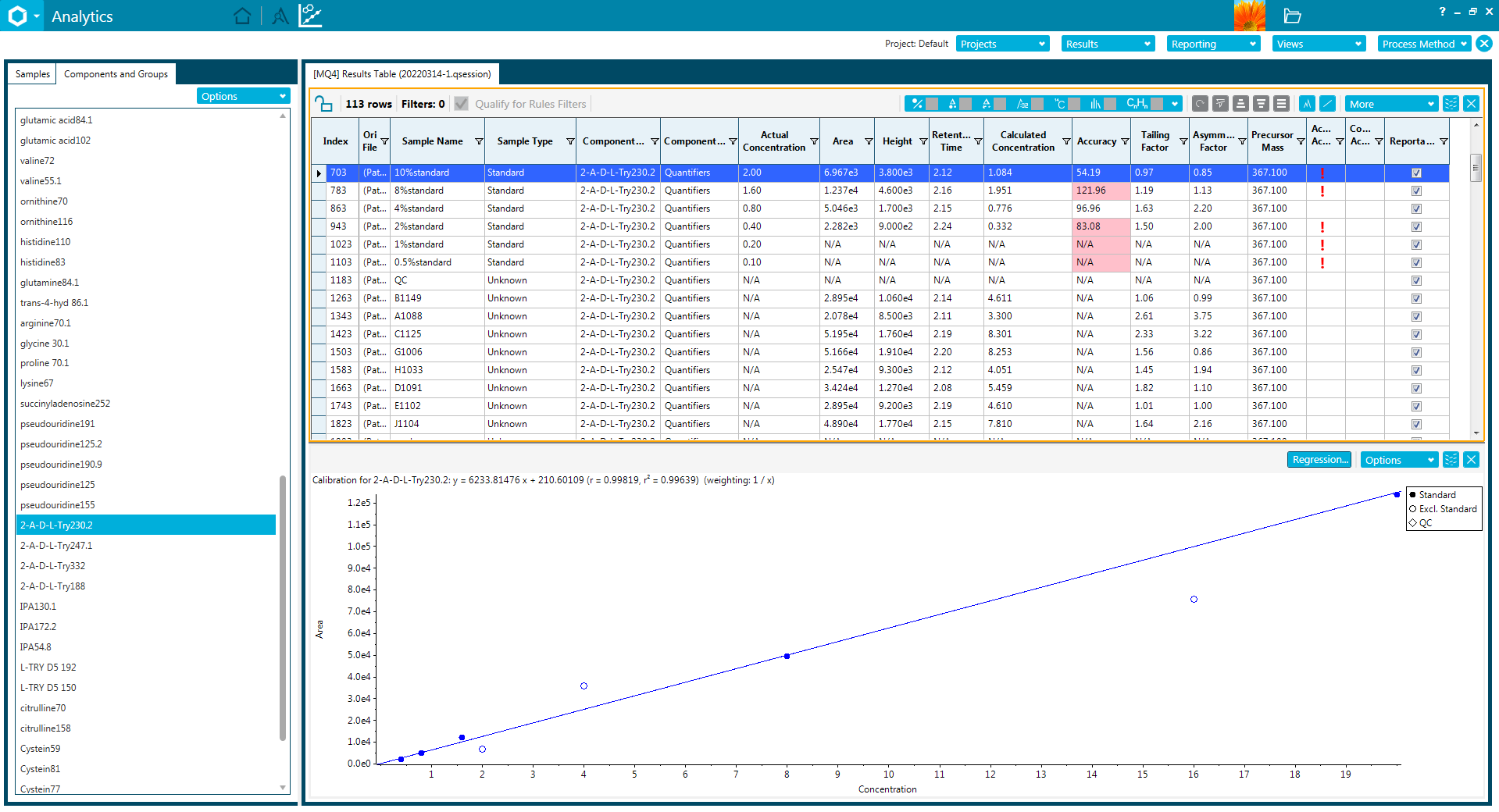


2-(α-D-Mannopyranosyl)-L-tryptophan R^2^=0.99639


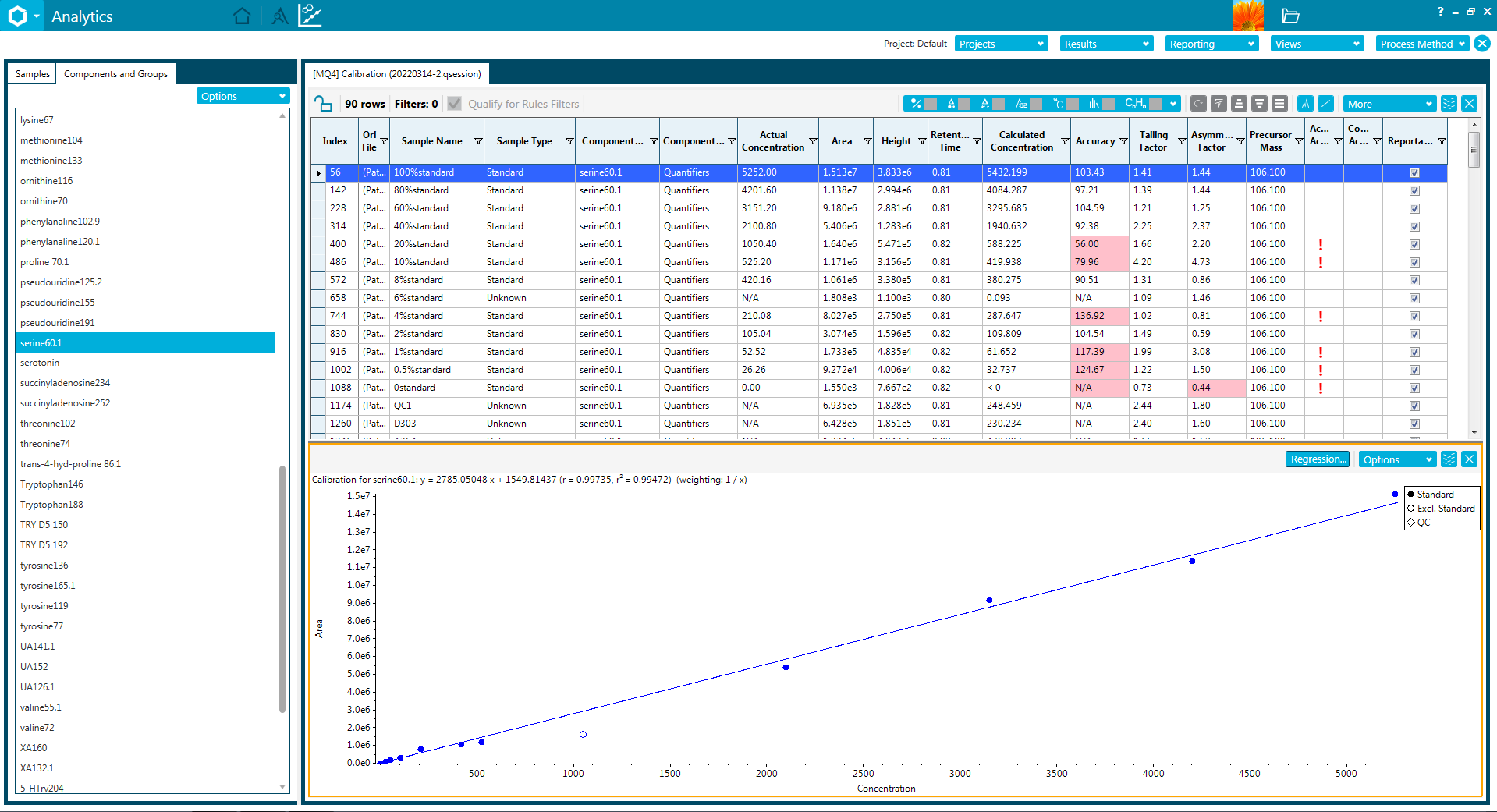


Serine R^2^=0.99472


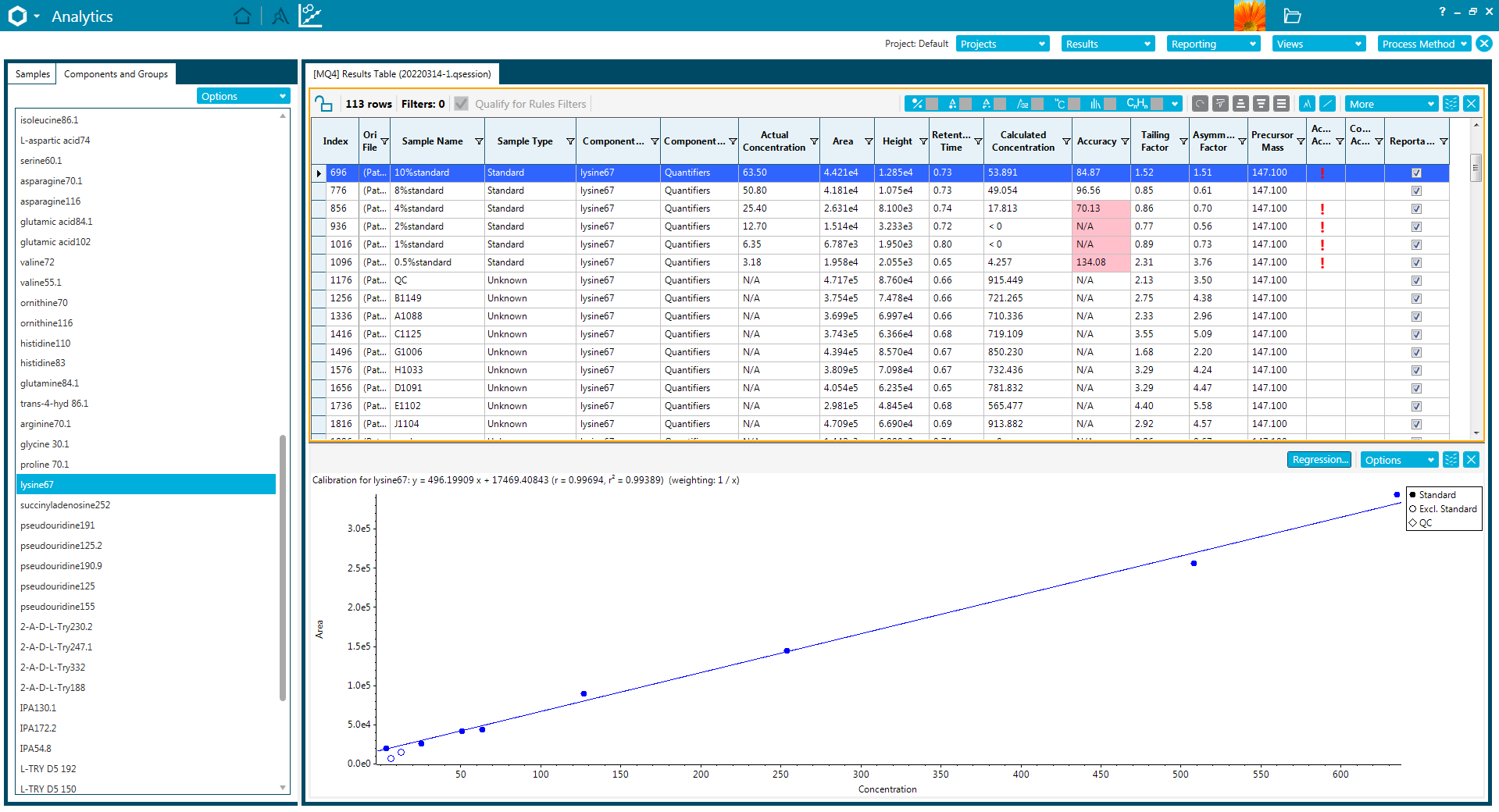


Lysine R^2^=0.99389


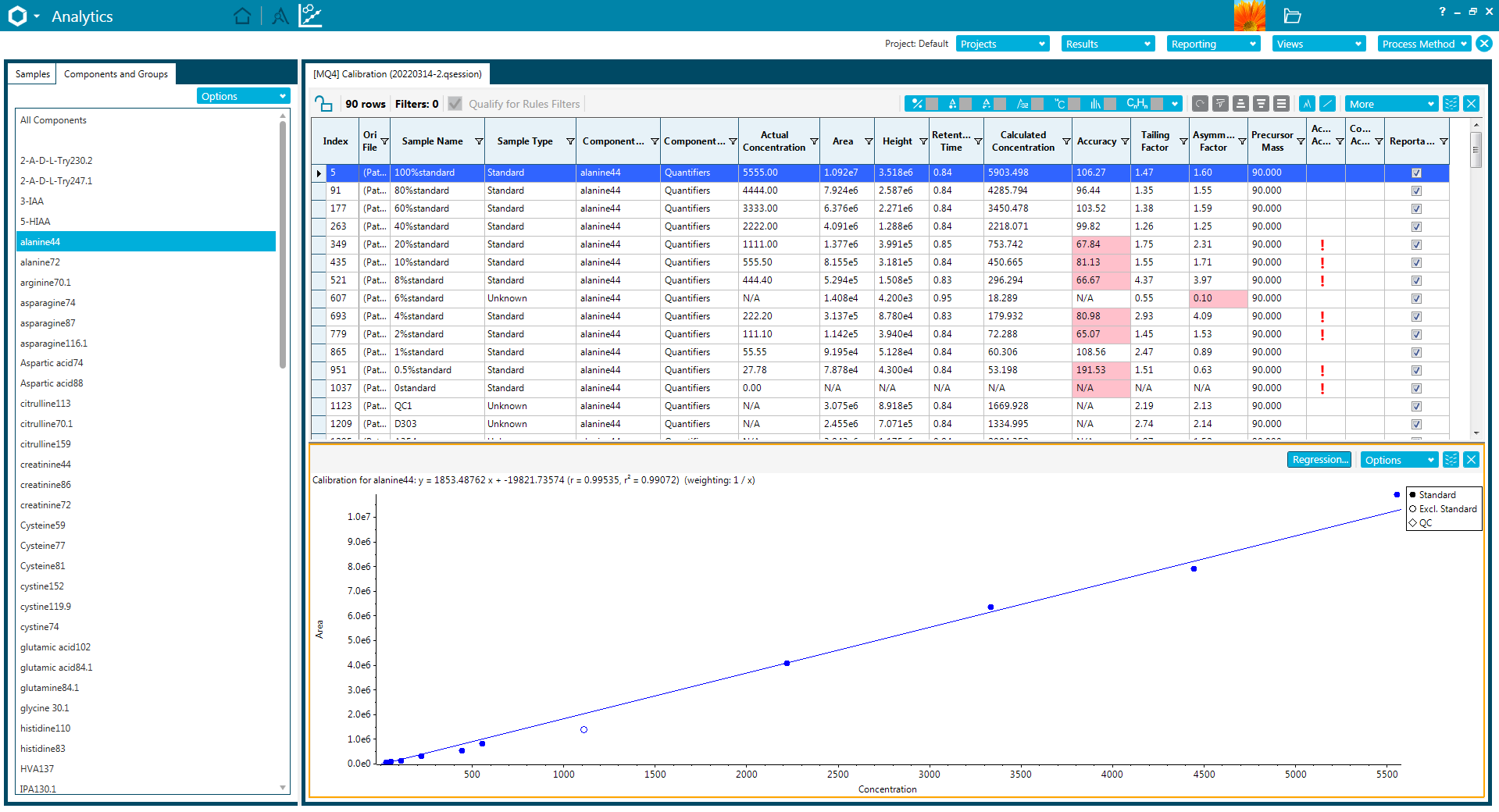


Alanine R^2^=0.99072


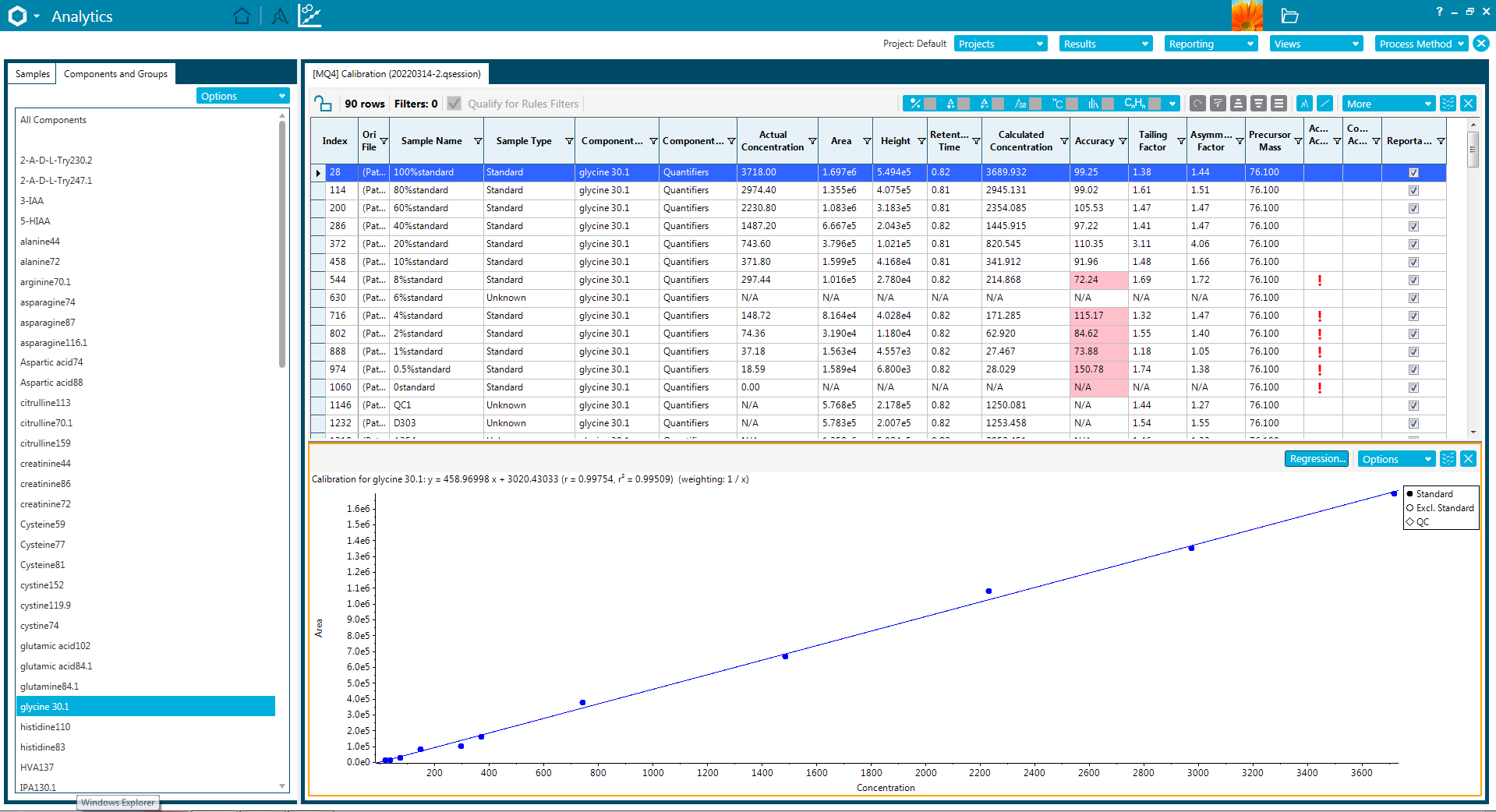


Glycine R^2^=0.99509


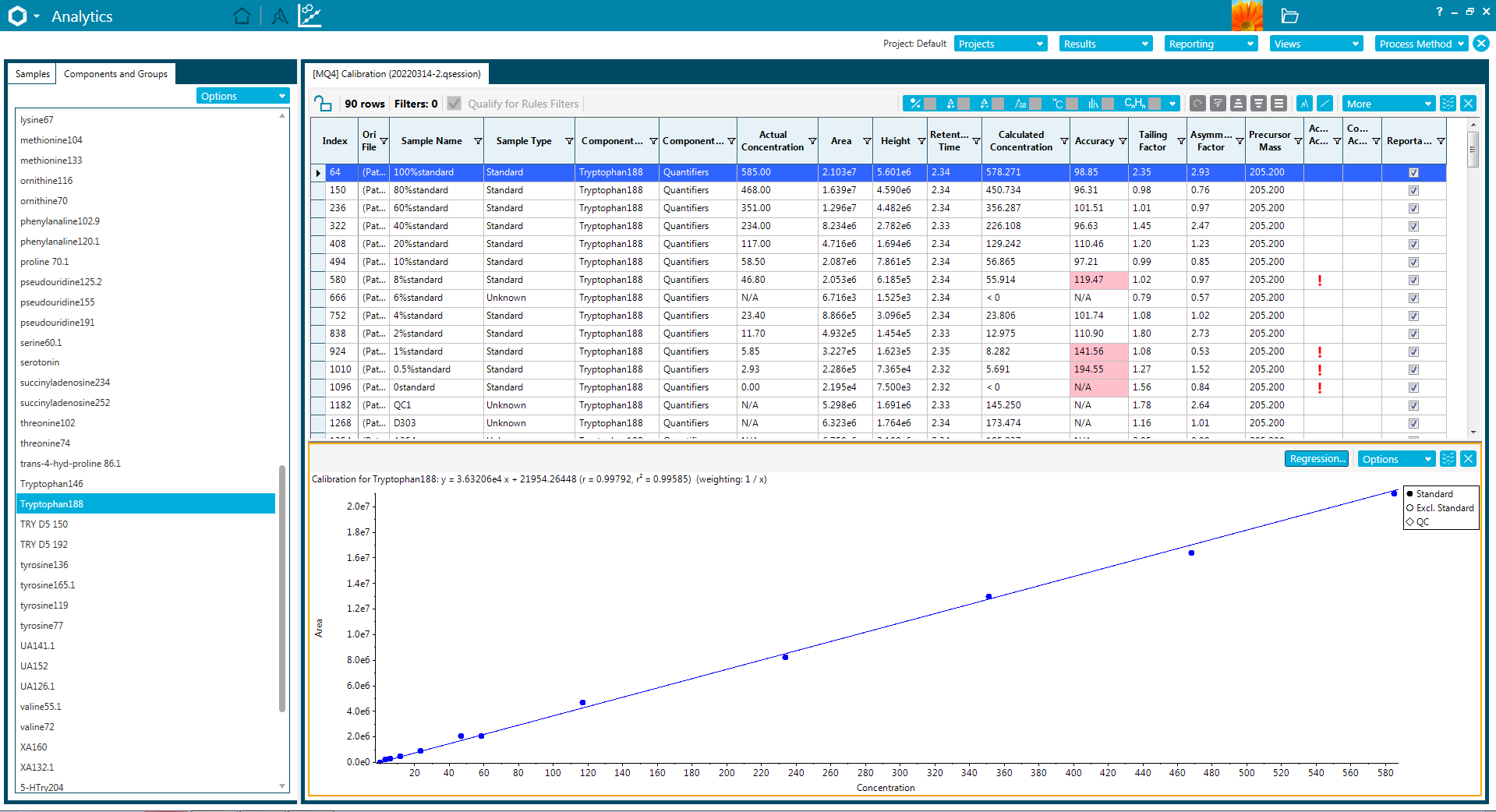


Tryptophan R^2^=0.99585


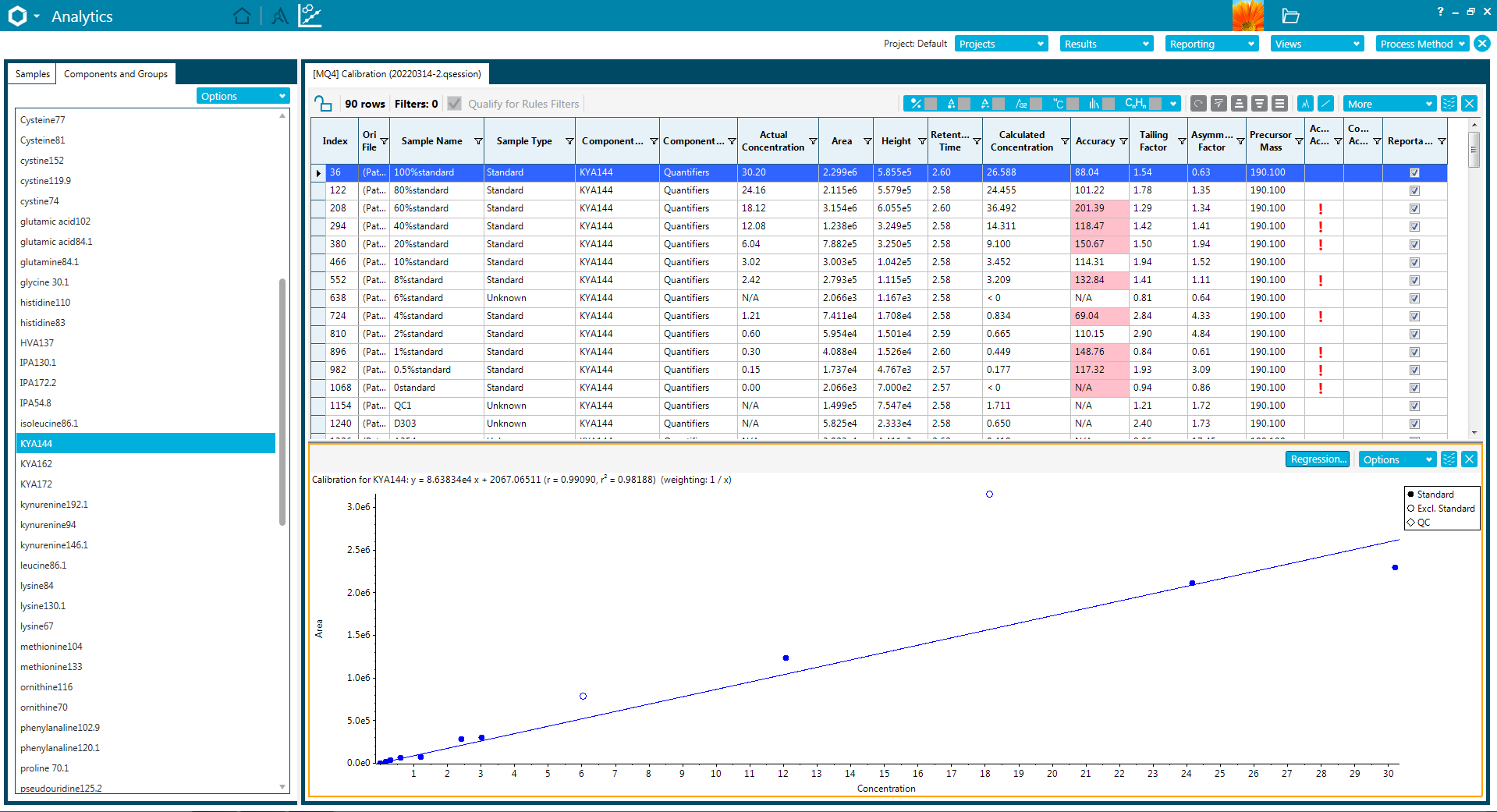


Kynurenic acid R^2^=0.98188


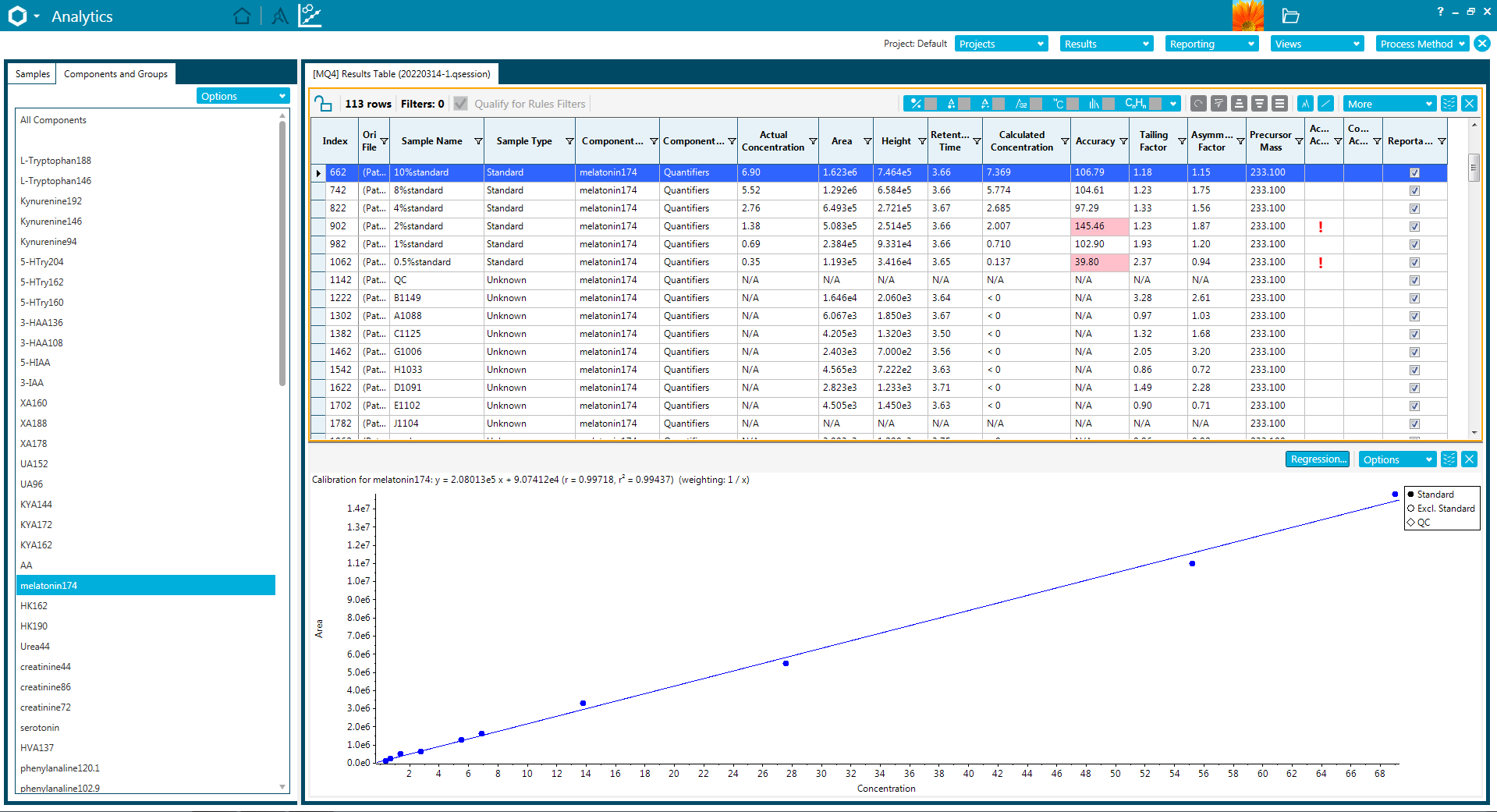


Melatonin R^2^=0.99348


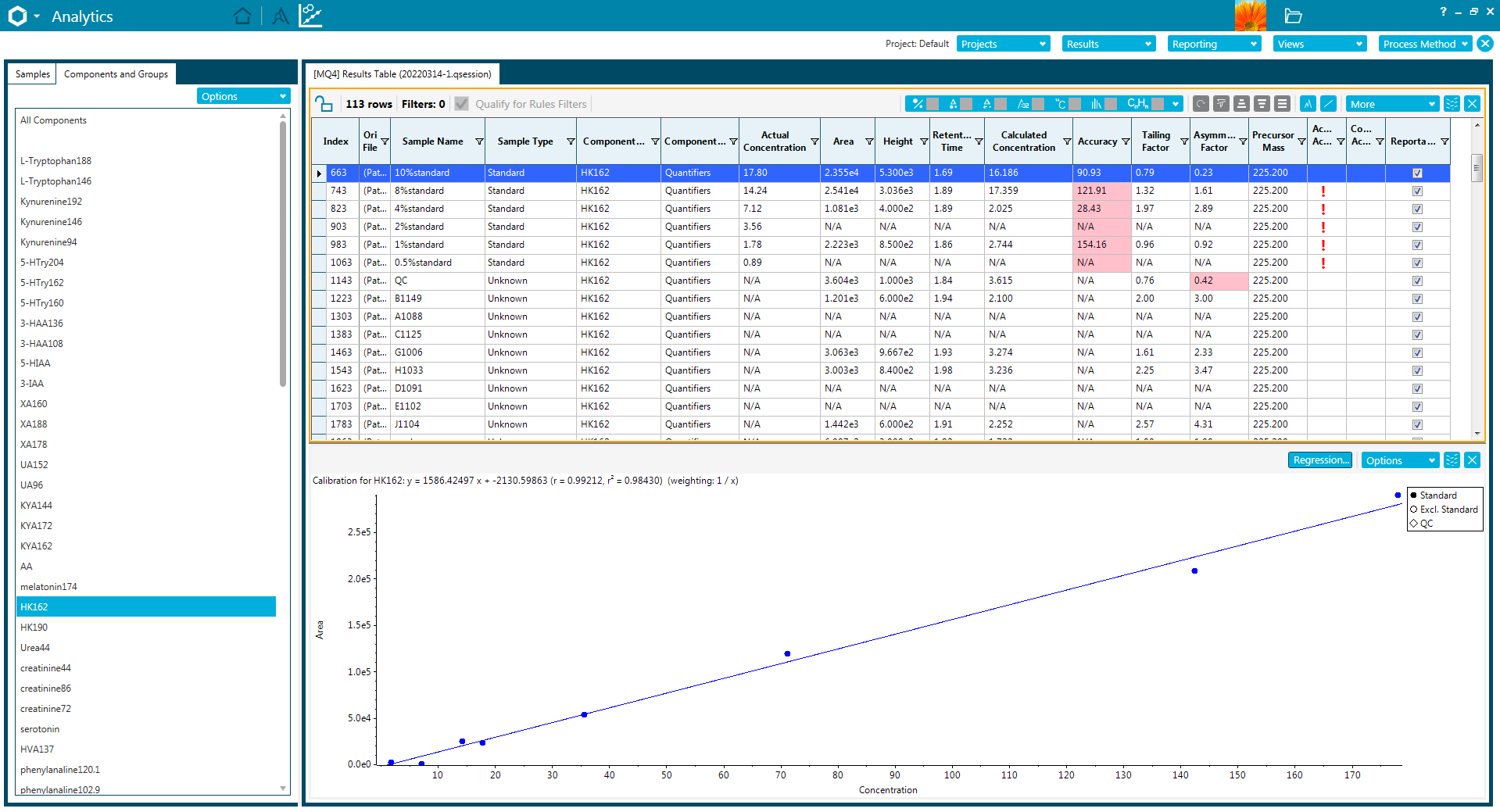


Hydroxykynurenine R^2^=0.98430


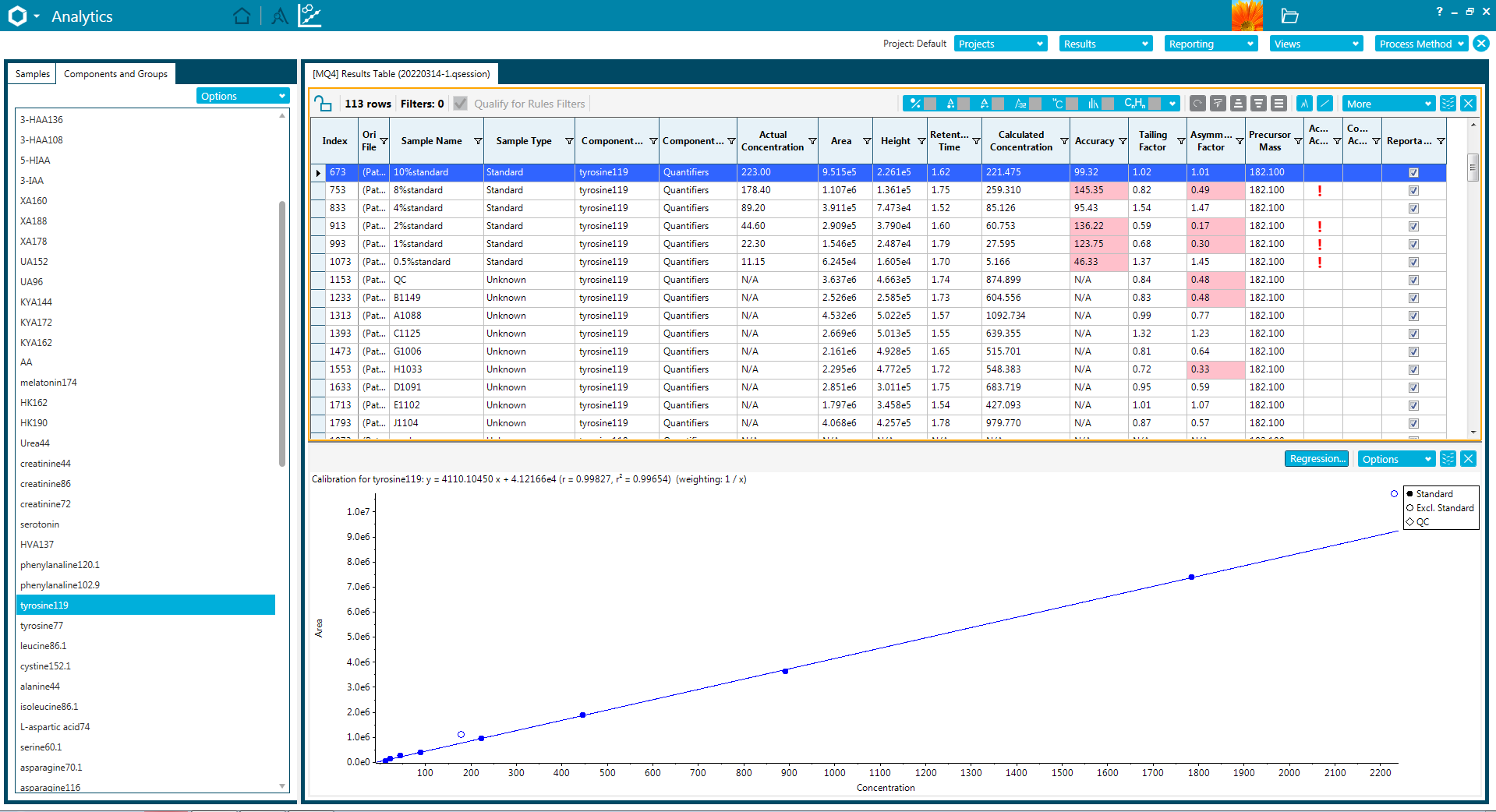


Tyrosine R^2^=0.99654


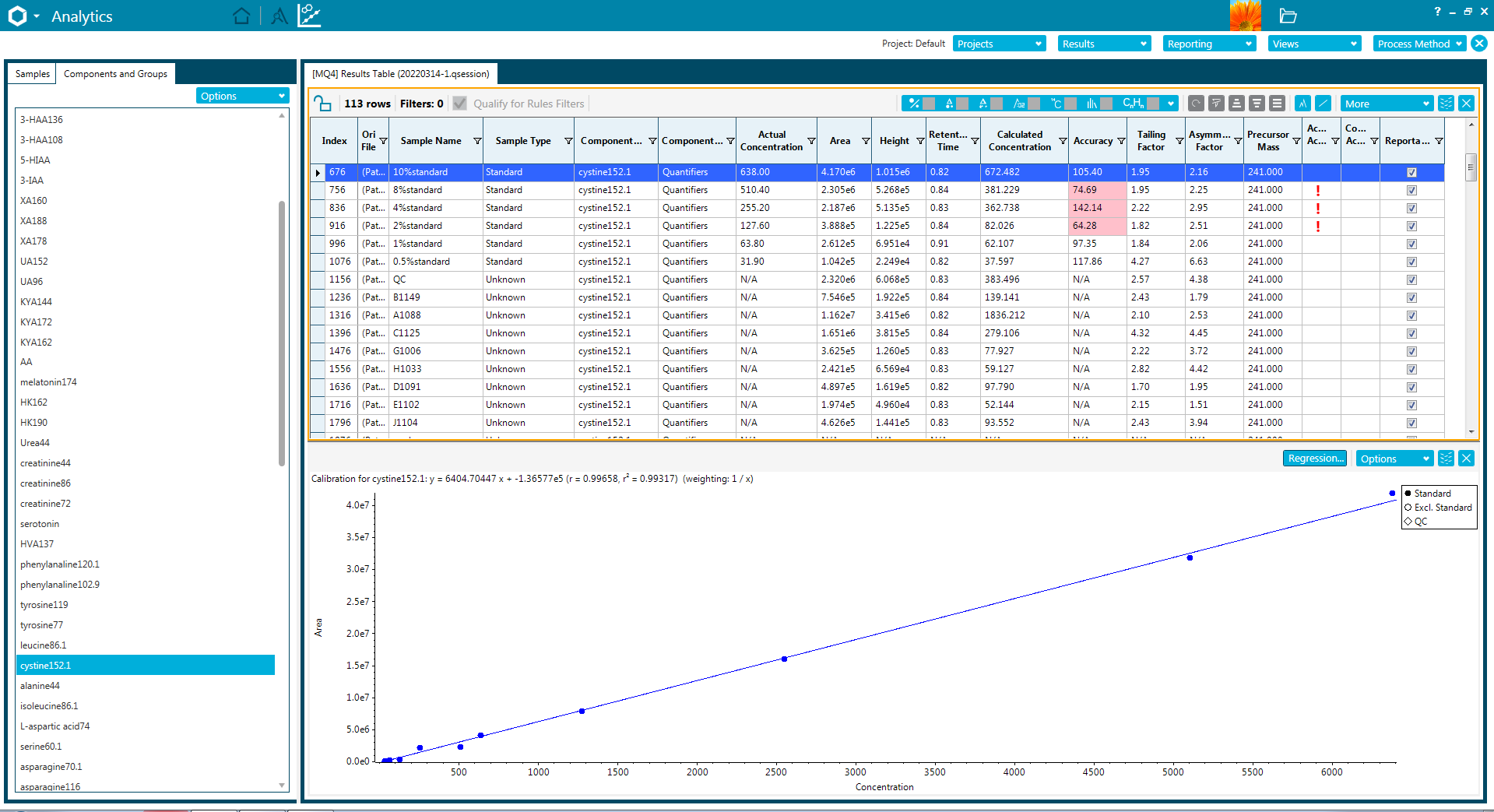


Cystine R^2^=0.99317


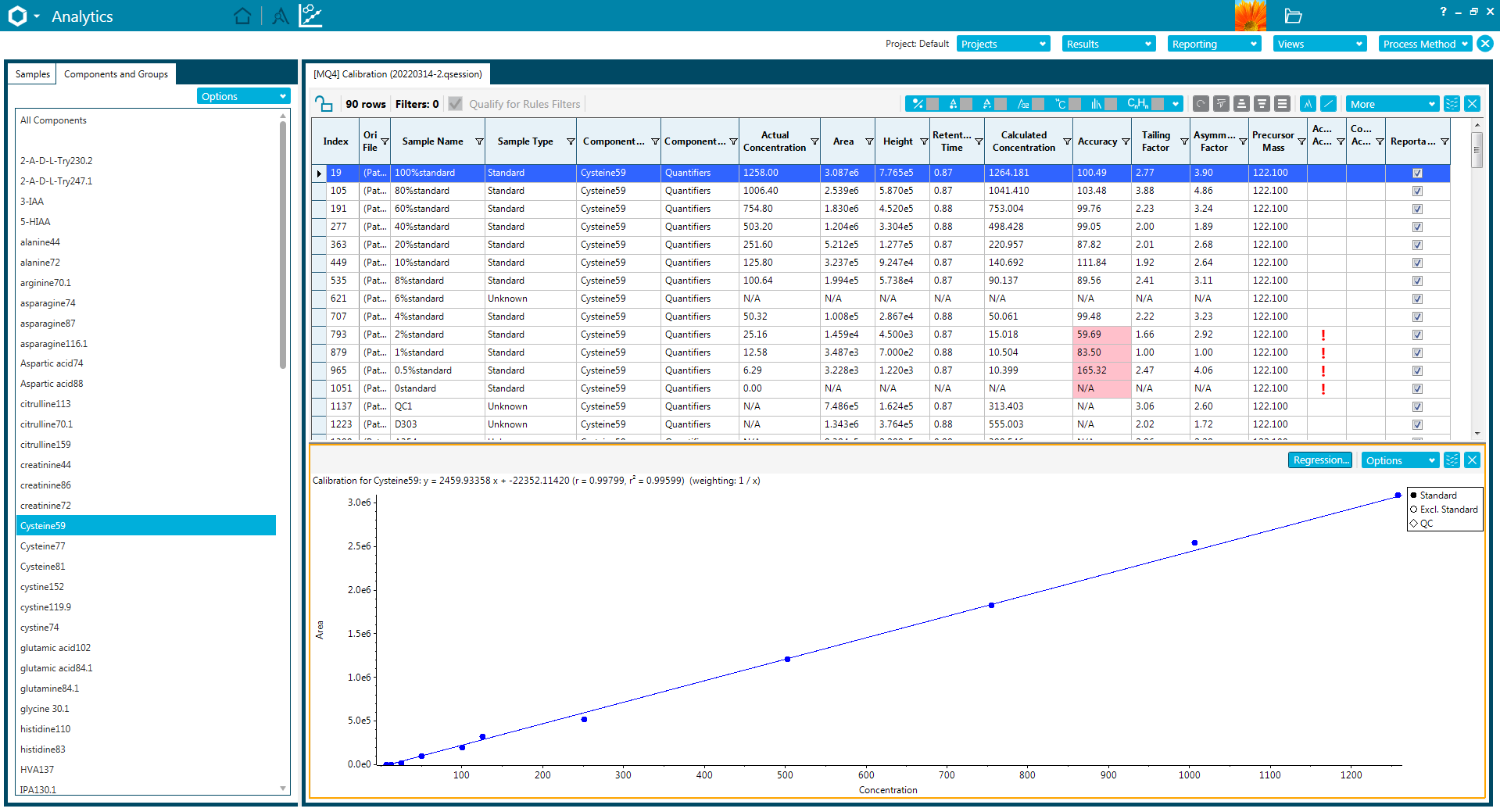


Cysteine R^2^=0.99599


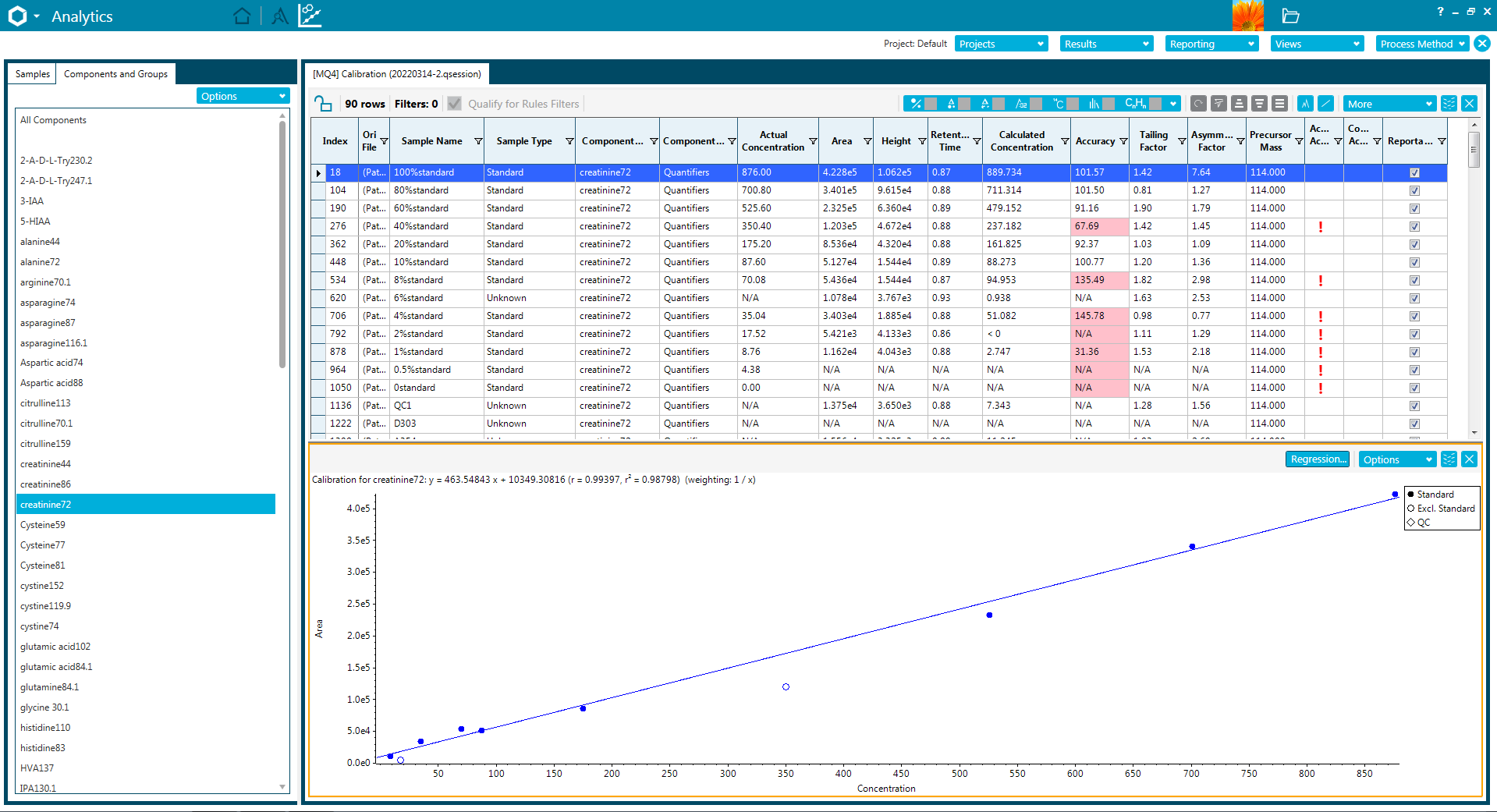


Creatinine R^2^=0.98798


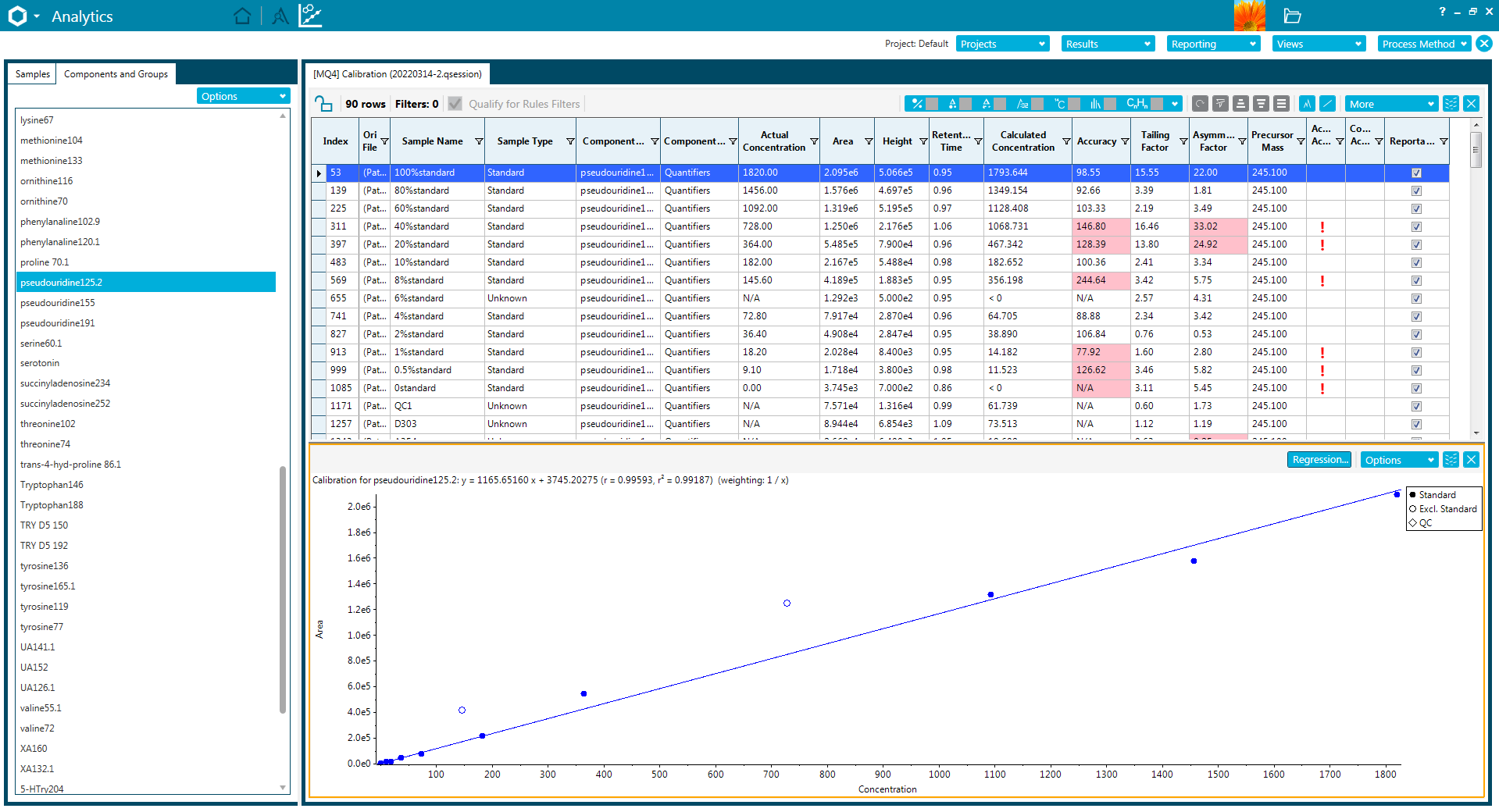


Pseudouridine R^2^=0.99187


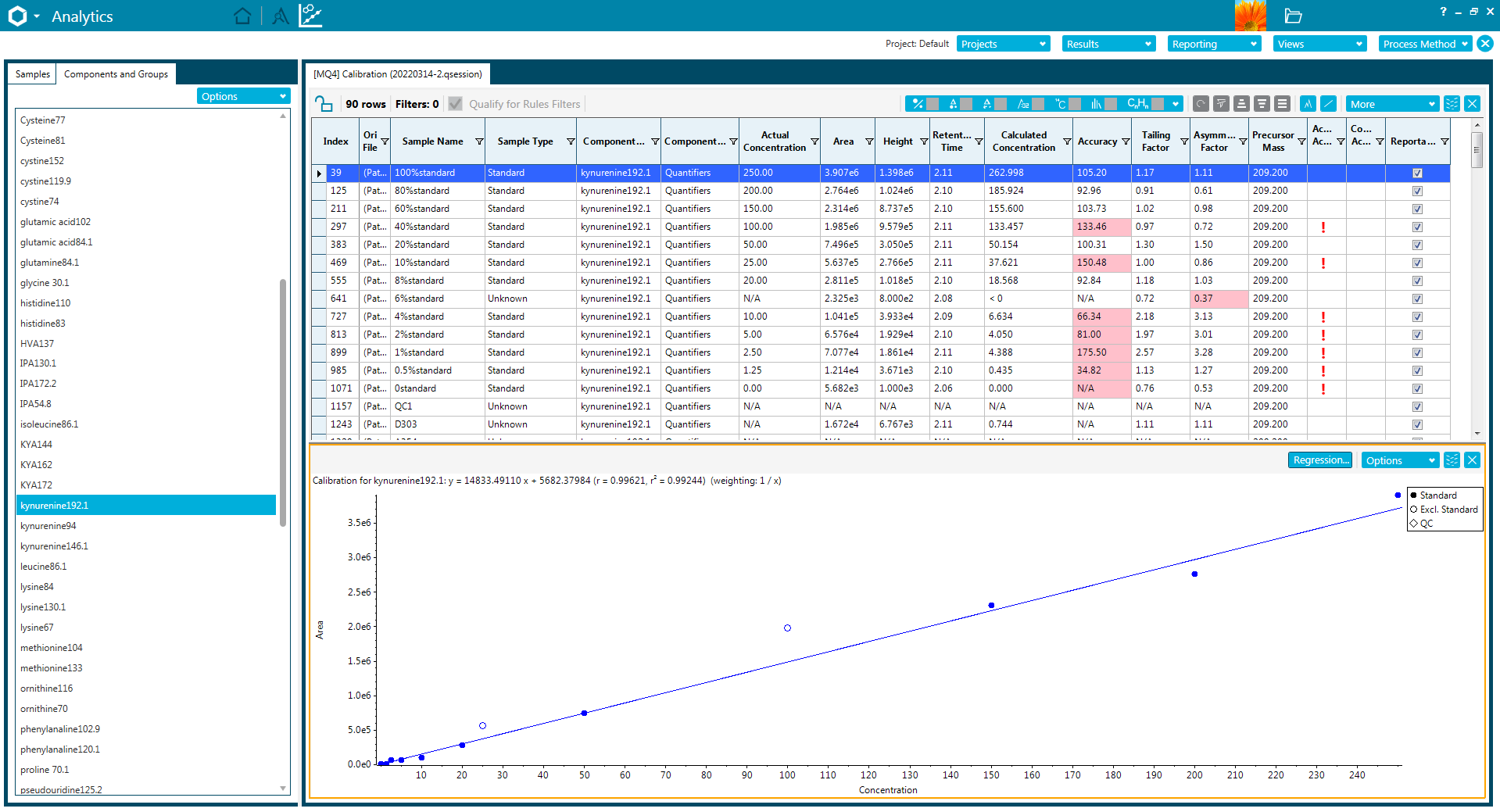


Kynurenine R^2^=0.99244

**Table S1**

**Q1/Q3 mass and MRM conditions for the selected metabolites.**

| **Metabolite** | **Q1 mass**  (Da) | **Q3 mass**  (Da) | **Dwelling Time**  (msec) | **Declustering Potential**  (volts) | **Collision Energy**  (volts) |
| --- | --- | --- | --- | --- | --- |
| MPT | 367.1 | 230.2 | 100 | 169.17 | 29.29 |
| Alanine | 90 | 44 | 100 | 29.31 | 21.28 |
| Asparagine | 133.1 | 74 | 100 | 57.76 | 20.97 |
| Aspartic acid | 134.1 | 74 | 100 | 45.21 | 18.75 |
| Citrulline | 176.1 | 159 | 100 | 66.06 | 13.51 |
| Creatinine | 114 | 44 | 100 | 77.54 | 22.49 |
| Cysteine | 122.1 | 59.1 | 100 | 60 | 29 |
| Cystine | 241.2 | 152 | 100 | 73.56 | 18.1 |
| Glutamic acid | 148.1 | 84.1 | 100 | 67.04 | 22.36 |
| Glutamine | 147.1 | 84.1 | 100 | 49.05 | 22.83 |
| Glycine | 76.1 | 30.1 | 100 | 31.68 | 20.38 |
| Histidine | 156.1 | 110 | 100 | 47.49 | 19.99 |
| Homovanillic acid | 183 | 137 | 100 | 103.08 | 25.25 |
| Isoleucine | 132.1 | 86.1 | 100 | 49.56 | 14.55 |
| Kynurenic acid | 190.1 | 144 | 100 | 53.15 | 28 |
| Kynurenine | 209.2 | 192.1 | 100 | 55.67 | 12.57 |
| Leucine | 132.001 | 86.1 | 100 | 58.81 | 16.19 |
| Lysine | 147.2 | 84 | 100 | 65.17 | 21.43 |
| Methionine | 150 | 104 | 100 | 70 | 14.5 |
| Ornithine | 133 | 70 | 100 | 45.33 | 24.58 |
| Phenylanaline | 166.1 | 120.1 | 100 | 78.96 | 30.86 |
| Proline | 116.1 | 70.1 | 100 | 74.97 | 21.38 |
| Pseudouridine | 245.1 | 191 | 100 | 108.61 | 19.97 |
| Serine | 106.1 | 60.1 | 100 | 50.78 | 15.44 |
| Serotonin | 177.1 | 160 | 100 | 44.32 | 17.48 |
| Succinyladenosine | 384.1 | 234 | 100 | 122.72 | 39.24 |
| Threonine | 120 | 74 | 100 | 50 | 13 |
| Trans-4-hydroxyproline | 132 | 86.1 | 100 | 66.95 | 19.66 |
| Tryptophan | 205.2 | 188 | 100 | 67.26 | 15.06 |
| Tyrosine | 182.1 | 136 | 100 | 61.97 | 18.53 |
| Uric acid | 169.1 | 141.1 | 100 | 121.31 | 24.62 |
| Valine | 118 | 72 | 100 | 61.36 | 17.24 |
| Xanthurenic acid | 206.1 | 132.1 | 100 | 82.52 | 40.04 |
| 5-hydroxytryptamine | 221 | 204 | 100 | 72.83 | 15.75 |
| 3-hydroxyanthranilic | 154.2 | 136 | 100 | 33.32 | 17.63 |
| Anthranilic acid | 138.1 | 120 | 100 | 60.72 | 18.26 |
| Melatonin | 233.1 | 174 | 100 | 69.81 | 25.67 |
| Hydroxykynurenine | 225 | 208.2 | 100 | 56.8 | 14.21 |
| L-TRYPTOPHAN (INDOLE-D5) | 210 | 192 | 100 | 61.85 | 16.69 |

**Table S2**

**Gradient diluted and corresponding concentrations of standards.**

|  | **100%**（ng/ml） | **80%**（ng/ml） | **60%**（ng/ml） | **40%**（ng/ml） | **20%**（ng/ml） | **10%**（ng/ml） | **8%**（ng/ml） | **6%**（ng/ml） | **4%**（ng/ml） | **2%**（ng/ml） | **1%**（ng/ml） |
| --- | --- | --- | --- | --- | --- | --- | --- | --- | --- | --- | --- |
| 5-hydroxytryptamine | 75 | 60 | 45 | 30 | 15 | 7.5 | 6 | 4.5 | 3 | 1.5 | 0.75 |
| Alanine | 5555 | 4444 | 3333 | 2222 | 1111 | 555.5 | 444.4 | 333.3 | 222.2 | 111.1 | 55.55 |
| Anthranilic acid | 23 | 18.4 | 13.8 | 9.2 | 4.6 | 2.3 | 1.84 | 1.38 | 0.92 | 0.46 | 0.23 |
| Arginine | 5208 | 4166.4 | 3124.8 | 2083.2 | 1041.6 | 520.8 | 416.64 | 312.48 | 208.32 | 104.16 | 52.08 |
| Asparagine | 620 | 496 | 372 | 248 | 124 | 62 | 49.6 | 37.2 | 24.8 | 12.4 | 6.2 |
| Asparitic acid | 2142 | 1713.6 | 1285.2 | 856.8 | 428.4 | 214.2 | 171.36 | 128.52 | 85.68 | 42.84 | 21.42 |
| Citrulline | 500 | 400 | 300 | 200 | 100 | 50 | 40 | 30 | 20 | 10 | 5 |
| Creatinine | 876 | 700.8 | 525.6 | 350.4 | 175.2 | 87.6 | 70.08 | 52.56 | 35.04 | 17.52 | 8.76 |
| Cysteine | 1258 | 1006.4 | 754.8 | 503.2 | 251.6 | 125.8 | 100.64 | 75.48 | 50.32 | 25.16 | 12.58 |
| Cystine | 6380 | 5104 | 3828 | 2552 | 1276 | 638 | 510.4 | 382.8 | 255.2 | 127.6 | 63.8 |
| Glutamic acid | 8415 | 6732 | 5049 | 3366 | 1683 | 841.5 | 673.2 | 504.9 | 336.6 | 168.3 | 84.15 |
| Glutamine | 5828 | 4662.4 | 3496.8 | 2331.2 | 1165.6 | 582.8 | 466.24 | 349.68 | 233.12 | 116.56 | 58.28 |
| Glycine | 3718 | 2974.4 | 2230.8 | 1487.2 | 743.6 | 371.8 | 297.44 | 223.08 | 148.72 | 74.36 | 37.18 |
| Histidine | 6880 | 5504 | 4128 | 2752 | 1376 | 688 | 550.4 | 412.8 | 275.2 | 137.6 | 68.8 |
| Homovanillic acid | 4934 | 3947.2 | 2960.4 | 1973.6 | 986.8 | 493.4 | 394.72 | 296.04 | 197.36 | 98.68 | 49.34 |
| Hydroxykynurenine | 178 | 142.4 | 106.8 | 71.2 | 35.6 | 17.8 | 14.24 | 10.68 | 7.12 | 3.56 | 1.78 |
| Isoleucine | 1268 | 1014.4 | 760.8 | 507.2 | 253.6 | 126.8 | 101.44 | 76.08 | 50.72 | 25.36 | 12.68 |
| Kynurenic acid | 30.2 | 24.16 | 18.12 | 12.08 | 6.04 | 3.02 | 2.416 | 1.812 | 1.208 | 0.604 | 0.302 |
| Kynurenine | 250 | 200 | 150 | 100 | 50 | 25 | 20 | 15 | 10 | 5 | 2.5 |
| Leucine | 8080 | 6464 | 4848 | 3232 | 1616 | 808 | 646.4 | 484.8 | 323.2 | 161.6 | 80.8 |
| Lysine | 635 | 508 | 381 | 254 | 127 | 63.5 | 50.8 | 38.1 | 25.4 | 12.7 | 6.35 |
| Melatonin | 69 | 55.2 | 41.4 | 27.6 | 13.8 | 6.9 | 5.52 | 4.14 | 2.76 | 1.38 | 0.69 |
| MPT | 20 | 16 | 12 | 8 | 4 | 2 | 1.6 | 1.2 | 0.8 | 0.4 | 0.2 |
| Methionine | 705.2 | 564.16 | 423.12 | 282.08 | 141.04 | 70.52 | 56.416 | 42.312 | 28.208 | 14.104 | 7.052 |
| Ornithine | 312 | 249.6 | 187.2 | 124.8 | 62.4 | 31.2 | 24.96 | 18.72 | 12.48 | 6.24 | 3.12 |
| Phenylanaline | 4680 | 3744 | 2808 | 1872 | 936 | 468 | 374.4 | 280.8 | 187.2 | 93.6 | 46.8 |
| Proline | 4852 | 3881.6 | 2911.2 | 1940.8 | 970.4 | 485.2 | 388.16 | 291.12 | 194.08 | 97.04 | 48.52 |
| Pseudouridine | 1820 | 1456 | 1092 | 728 | 364 | 182 | 145.6 | 109.2 | 72.8 | 36.4 | 18.2 |
| Serine | 5252 | 4201.6 | 3151.2 | 2100.8 | 1050.4 | 525.2 | 420.16 | 315.12 | 210.08 | 105.04 | 52.52 |
| Serotonin | 550 | 440 | 330 | 220 | 110 | 55 | 44 | 33 | 22 | 11 | 5.5 |
| succinyladenosine | 120 | 96 | 72 | 48 | 24 | 12 | 9.6 | 7.2 | 4.8 | 2.4 | 1.2 |
| Threonine | 1188 | 950.4 | 712.8 | 475.2 | 237.6 | 118.8 | 95.04 | 71.28 | 47.52 | 23.76 | 11.88 |
| T-H-proline | 1338.5 | 1070.8 | 803.1 | 535.4 | 267.7 | 133.85 | 107.08 | 80.31 | 53.54 | 26.77 | 13.385 |
| Tryptophan | 585 | 468 | 351 | 234 | 117 | 58.5 | 46.8 | 35.1 | 23.4 | 11.7 | 5.85 |
| Tyrosine | 2230 | 1784 | 1338 | 892 | 446 | 223 | 178.4 | 133.8 | 89.2 | 44.6 | 22.3 |
| Urea | 10250 | 8200 | 6150 | 4100 | 2050 | 1025 | 820 | 615 | 410 | 205 | 102.5 |
| Uric acid | 2870 | 2296 | 1722 | 1148 | 574 | 287 | 229.6 | 172.2 | 114.8 | 57.4 | 28.7 |
| Valine | 2364 | 1891.2 | 1418.4 | 945.6 | 472.8 | 236.4 | 189.12 | 141.84 | 94.56 | 47.28 | 23.64 |
| Tryptophan indole D5 | 1740 | 1392 | 1044 | 696 | 348 | 174 | 139.2 | 104.4 | 69.6 | 34.8 | 17.4 |

**Table S3**

**Detected concentrations of metabolites in T1DM mice.**

| **Metabolite** | **T1D-ctrl**  **(ng/ml)** | **STZ**  **(ng/ml)** | **STZ+Art**  **(ng/ml)** |
| --- | --- | --- | --- |
| **Alanine** | 1553.41±129.84 | 1723.42±177.24 | 1959.07±342.39 |
| **Arginine** | 388.29±66.43 | 332.28±81.85 | 300.43±63.42 |
| **Asparagine** | 127.75±24.93 | 116.85±18.45 | 131.96±17.21 |
| **Aspartic acid** | 4976.83±851.46 | 3206.01±961.49*** | 3641.53±475.88 |
| **Citrulline** | 60.86±11.66 | 76.53±13.56* | 57.49±12.43## |
| **Creatinine** | 21.23±4.52 | 27.83±11.36 | 29.31±2.62 |
| **Cysteine** | 570.92±260.03 | 654.32±264.33 | 1502.95±1340.21 |
| **Cystine** | 1523.89±1434.72 | 1017.55±1058.48 | 1140.75±1198.55 |
| **Glutamic acid** | 7455.07±918.08 | 5717.74±857.24** | 6101.23±1236.04 |
| **Glutamine** | 2338.59±375.98 | 1551.96±91.16*** | 1838.26±269.57# |
| **Glycine** | 4941.24±1022.76 | 3381.03±1235.67* | 4261.18±2325.59 |
| **Histidine** | 392.65±66.83 | 281.05±39.19*** | 319.08±55.88 |
| **IPA** | 9.23±2.71 | 7.89±3.53 | 3.23±1.4## |
| **Isoleucine** | 124.93±29.5 | 191.64±27.38*** | 134.87±41.54## |
| **KYA** | 1.1±1.67 | 1.32±0.81 | 0.77±0.44 |
| **Leucine** | 1362.23±364.48 | 1706.84±557.39 | 1345.11±431.89 |
| **Lysine** | 887.59±170.97 | 780.1±127.77 | 714.15±140.34 |
| **Methionine** | 562.65±310.63 | 426.48±197.99 | 410.58±250.32 |
| **MPT** | 5.45±1.42 | 6.25±1.36 | 5.46±1.08 |
| **Ornithine** | 86.83±17.52 | 77.38±12.18 | 84.7±12.77 |
| **Phenylanaline** | 348.94±70.39 | 256.88±97.02* | 293.03±70.26 |
| **Proline** | 2489.21±276.41 | 2874.2±694.81 | 2551.46±399.78 |
| **Pseudouridine** | 27.07±40.36 | 15.23±7.74 | 15.16±5.02 |
| **Serine** | 290.22±38.98 | 267.52±41.91 | 292.27±65.65 |
| **Serotonin** | 75.11±31.42 | 73.11±17.28 | 53.36±18.85 |
| **T-H-proline** | 532.33±476.77 | 749.42±618.63 | 607.8±471.39 |
| **Threonine** | 378.06±50.19 | 356.14±64.5 | 401.68±58.79 |
| **Tryptophan** | 147.26±58.47 | 155.18±17.61 | 153.9±43.42 |
| **Tyrosine** | 833.53±163.35 | 843.11±164.98 | 802.09±345.61 |
| **Urea** | 13231.56±3664.37 | 11709.34±2347.57 | 11366.02±1868.51 |
| **Valine** | 613.82±70.62 | 836.66±56.8*** | 630.84±215.16 |
| ***Total protein*** | *5.85±0.26(mg/ml)* | *5.24±0.78(mg/ml)* | *5.29±1.87(mg/ml)* |
| ***Tryptophan-D5*** | *1.69±0.11(ug/ml)* | *1.71±0.08(ug/ml)* | *1.64±0.22(ug/ml)* |

For each group, n=6-8. * *P*<0.05, ** *P*<0.01 and *** *P*<0.001 vs. the Ctrl group. # *P*<0.05 and ## *P*<0.01vs. the STZ group.

**Table S4**

**Dectected concentrations of metabolites in T2DM mice.**

| **Metabolite** | **T2D-ctrl**  **(ng/ml)** | **db/db**  **(ng/ml)** | **db+Art**  **(ng/ml)** |
| --- | --- | --- | --- |
| **5-Hydroxytryptamine** | 0.77±0.23 | 0.69±0.38 | 0.58±0.29 |
| **AA** | 0.13±0.02 | 0.15±0.1 | 0.15±0.04 |
| **Alanine** | 1625.04±394.12 | 1545.46±510.54 | 1928.67±179.7 |
| **Arginine** | 450.75±106.71 | 346.86±92.56 | 359.14±106.23 |
| **Asparagine** | 131.88±16.05 | 146.41±14.9 | 159.76±34 |
| **Aspartic acid** | 2033.52±384.64 | 969.76±332.17*** | 1432.26±246.41## |
| **Citrulline** | 22.22±6.05 | 40.8±15.62* | 23.11±3.72 |
| **Cysteine** | 360.56±236.19 | 392.76±223.93 | 220.07±112.26 |
| **Glutamic acid** | 5797.83±537.67 | 4877.12±1105.02* | 4425.75±633.18 |
| **Glycine** | 2459.26±456.88 | 1187.43±277.15*** | 1102.08±191.42 |
| **Histidine** | 329.67±74.53 | 259.57±47.38* | 282.87±53.02 |
| **HVA** | 1325.81±442.88 | 1149.81±234.79 | 1273±344.63 |
| **Isoleucine** | 182.55±41.43 | 210.65±49.23 | 193.57±49.14 |
| **KYA** | 0.93±0.51 | 2.04±2.71 | 1.09±1.28 |
| **Leucine** | 1330.73±465.49 | 1183.55±266.58 | 1076.02±345.63 |
| **Lysine** | 262.09±54.66 | 188.22±5.7* | 184.24±13.54 |
| **MPT** | 4.67±1.6 | 7.68±5.67 | 5.42±2.93 |
| **Ornithine** | 72.18±11.84 | 59.28±10.2* | 57.06±12.47 |
| **Phenylanaline** | 463.26±168.04 | 497.62±99.09 | 535.34±140.54 |
| **Proline** | 3435.71±699.2 | 3134.05±476.6 | 3394.73±556.63 |
| **Serine** | 387.31±85.22 | 236.06±43.15** | 284.9±32.11 |
| **Serotonin** | 84.05±24.22 | 48.98±10.35* | 38.53±16.56 |
| **Succinyladenosine** | 19.58±4.66 | 43.19±27.48 | 28.08±5.69 |
| **T-H-proline** | 185.47±47.08 | 209.61±40.59 | 195.17±47.6 |
| **Threonine** | 344.24±56.55 | 272.45±51.96** | 325.32±31.54# |
| **Tryptophan** | 182.23±48.52 | 158.61±21.18 | 175.57±38.03 |
| **Tyrosine** | 747.45±276.28 | 576.94±166.36 | 650.1±141.06 |
| **UA** | 84.74±20.03 | 103.03±42.58 | 141.75±60.42 |
| **Valine** | 668.2±174.17 | 696.34±208.57 | 648.62±129.55 |
| ***Total Protein*** | *7.71±0.45(mg/ml)* | *6.84±0.71(mg/ml)* | *7.17±0.66(mg/ml)* |
| ***Tryptophan-D5*** | *1.65±0.14(ug/ml)* | *1.59±0.16(ug/ml)* | *1.76±0.1(ug/ml)* |

For each group, n=6-8. * *P*<0.05, ** *P*<0.01 and *** *P*<0.001 vs. the Ctrl group. # *P*<0.05 and ## *P*<0.01vs. the db/db group.

**Table S5**

**Metabolites content adjusted by total protein in T1DM mice.**

| **Metabolite** | **T1D-ctrl**  **(ng/mg)** | **STZ**  **(ng/mg)** | **STZ+Art**  **(ng/mg)** |
| --- | --- | --- | --- |
| **Alanine** | 265.88±25.55 | 337.81±77.65* | 325.32±63.35 |
| **Arginine** | 66.56±12.46 | 64.69±18.27 | 50.04±12.27# |
| **Asparagine** | 21.87±4.42 | 22.74±4.96 | 21.89±3.32 |
| **Aspartic acid** | 850.47±143.38 | 621.23±187.97** | 601.58±73.26 |
| **Citrulline** | 10.42±2.07 | 14.78±2.7*** | 9.56±2.36### |
| **Creatinine** | 3.62±0.68 | 5.29±1.95 | 4.87±0.61 |
| **Cysteine** | 97.29±43.84 | 128.58±57.18 | 255.07±239.4 |
| **Cystine** | 262.93±251.36 | 216.06±251.57 | 191.95±202.57 |
| **Glutamic acid** | 1274.57±154.71 | 1129.44±349.03 | 1015.42±238.13 |
| **Glutamine** | 401.24±75.39 | 304.19±65.45** | 304.72±49.07 |
| **Glycine** | 846.56±189.34 | 671.88±311.43 | 712.55±407.43 |
| **Histidine** | 67.46±13.28 | 54.8±11.72* | 53.21±11.53 |
| **IPA** | 1.58±0.46 | 1.58±0.86 | 0.53±0.24## |
| **Isoleucine** | 21.46±5.7 | 37.05±5.97*** | 22.48±7.59### |
| **KYA** | 0.2±0.31 | 0.26±0.16 | 0.13±0.08 |
| **Leucine** | 234.06±68.15 | 325.76±88.77* | 223.07±75.28# |
| **Lysine** | 152.09±30.71 | 154.91±52.54 | 119.18±28.5 |
| **Methionine** | 96.12±52.16 | 87±54.65 | 69.79±46.87 |
| **MPT** | 0.94±0.28 | 1.21±0.29* | 0.91±0.21# |
| **Ornithine** | 14.85±3.01 | 15.12±3.65 | 14.09±2.65 |
| **Phenylanaline** | 59.65±11.93 | 50.66±20.56 | 48.8±12.76 |
| **Proline** | 426.84±58.84 | 548.38±95.18** | 425.57±87.93## |
| **Pseudouridine** | 4.83±7.54 | 2.93±1.41 | 2.54±0.95 |
| **Serine** | 49.67±7.24 | 52.14±11.31 | 48.55±11.76 |
| **Serotonin** | 12.74±5.15 | 14.34±4.5 | 8.74±2.89# |
| **T-H-proline** | 92.27±83.82 | 149.63±124.2 | 102.9±80.05 |
| **Threonine** | 64.82±10.17 | 69.22±15.51 | 66.75±11.34 |
| **Tryptophan** | 25.11±9.85 | 30.1±4.78 | 25.8±8.42 |
| **Tyrosine** | 142.67±28.51 | 164.11±41.55 | 134.21±60.83 |
| **Urea** | 2277.69±717.17 | 2291.3±629.43 | 1890.4±383.53 |
| **Valine** | 105.22±14.98 | 162.86±28.07*** | 105.69±39.31### |

For each group, n=6-8. * *P*<0.05, ** *P*<0.01 and *** *P*<0.001 vs. the Ctrl group. # *P*<0.05, ## *P*<0.01 and ### *P*<0.001 vs. the STZ group.

**Table S6**

**Metabolite content adjusted by total protein in T2DM mice.**

| **Metabolite** | **T2D-ctrl （ng/mg）** | **db/db（ng/mg）** | **db+Art（ng/mg）** |
| --- | --- | --- | --- |
| **5-Hydroxytryptamine** | 0.1±0.03 | 0.1±0.05 | 0.08±0.05 |
| **AA** | 0.02±0 | 0.02±0.02 | 0.02±0.01 |
| **Alanine** | 212.26±56.06 | 224.36±62.89 | 270.8±31.19 |
| **Arginine** | 58.93±16.06 | 51.84±17.22 | 51.46±19.85 |
| **Asparagine** | 17.13±2.26 | 21.74±4.18 | 22.9±8.09 |
| **Aspartic acid** | 265.02±56.76 | 141.55±43.91*** | 201.73±40.79# |
| **Citrulline** | 2.91±0.89 | 6.2±3.03 | 3.26±0.66 |
| **Cysteine** | 47.62±31.79 | 57.4±32.38 | 30.28±14.39# |
| **Glutamic acid** | 756.05±105.1 | 710.98±148.52 | 627.92±143.41 |
| **Glycine** | 321.67±72.4 | 173.55±36.03*** | 155.77±35.71 |
| **Histidine** | 43.29±11.92 | 38.24±7.75 | 39.93±9.18 |
| **HVA** | 172.94±60.01 | 170.64±42.22 | 179.02±50.02 |
| **Isoleucine** | 23.72±5.47 | 30.92±7.1* | 27.18±6.98 |
| **KYA** | 0.12±0.06 | 0.32±0.47 | 0.17±0.24 |
| **Leucine** | 173.63±64.34 | 175.05±47.16 | 154.29±66.67 |
| **Lysine** | 34.34±8.83 | 27.77±2.95 | 25.91±3.2 |
| **MPT** | 0.61±0.23 | 1.21±1.04 | 0.8±0.57 |
| **Ornithine** | 9.41±1.73 | 8.89±2.55 | 8.09±2.27 |
| **Phenylanaline** | 59.86±21.54 | 73.79±17.74 | 76.41±27.2 |
| **Proline** | 447.32±98.97 | 466.13±114.22 | 483.35±135.03 |
| **Serine** | 50.75±13.24 | 34.56±5.32* | 40.31±7.81 |
| **Serotonin** | 11±3.41 | 7.21±1.63* | 5.5±2.73 |
| **Succinyladenosine** | 2.56±0.67 | 6.44±4.19 | 3.95±0.87 |
| **T-H-proline** | 24.11±6.51 | 31.04±7.5 | 27.7±8.46 |
| **Threonine** | 44.97±9.06 | 39.99±7.61 | 45.97±8.09 |
| **Tryptophan** | 23.85±7.04 | 23.34±3.62 | 24.97±7.61 |
| **Tyrosine** | 97.54±37.63 | 83.99±21.44 | 91.66±22.7 |
| **UA** | 10.93±2.11 | 15.72±8.25 | 20.51±11.52 |
| **Valine** | 87.21±24.94 | 102.16±30.99 | 90.99±18.44 |

For each group, n=6-8. * *P*<0.05 and *** *P*<0.001 vs. the Ctrl group. # *P*<0.05 vs. the db/db group.

**Table S7**

**Metabolites corrected by total protein and tryptophan indole D5 in T1DM mice.**

| **Metabolite** | **T1D-ctrl**  **(ng/mg/ug/ml)** | **STZ (ng/mg/ug/ml)** | **STZ+Art**  **(ng/mg/ug/ml)** |
| --- | --- | --- | --- |
| **Alanine** | 158.75±21.95 | 198.51±51.47 | 200.15±38.35 |
| **Arginine** | 39.67±7.95 | 37.73±10.44 | 30.87±7.9 |
| **Asparagine** | 12.93±2.29 | 13.29±2.96 | 13.51±2.32 |
| **Aspartic acid** | 502.15±56.94 | 363.29±111.62** | 370.41±48.29 |
| **Citrulline** | 6.16±1.03 | 8.64±1.63** | 5.97±1.91## |
| **Creatinine** | 2.15±0.42 | 3.09±1.18* | 3.02±0.62 |
| **Cystein** | 58.12±27.97 | 75.22±33.93 | 154.47±142.79 |
| **Cystine** | 154.2±148.15 | 125.42±149.28 | 116.89±124.59 |
| **Glutamic acid** | 756.03±76.93 | 661.11±213.68 | 625.18±149.18 |
| **Glutamine** | 238.79±47.14 | 178.31±41.87** | 187.71±31.8 |
| **Glycine** | 503.5±112.43 | 391.92±183.56 | 434.47±245.28 |
| **Histidine** | 40.14±7.95 | 32.15±7.67* | 32.76±7.17 |
| **IPA** | 0.94±0.29 | 0.93±0.52 | 0.33±0.15## |
| **Isoleucine** | 12.82±3.61 | 21.67±3.69** | 13.67±4.32## |
| **KYA** | 0.12±0.19 | 0.15±0.09 | 0.08±0.04 |
| **Leucine** | 138.85±41.28 | 189.95±49.53* | 136.26±44.72# |
| **Lysine** | 90.78±20.29 | 91.15±33.32 | 73.31±17.35 |
| **Methionine** | 57.91±33.04 | 51.46±33.72 | 42.5±28.98 |
| **MPT** | 0.56±0.17 | 0.71±0.18 | 0.56±0.14 |
| **Ornithine** | 8.8±1.69 | 8.84±2.17 | 8.72±1.89 |
| **Phenylanaline** | 35.41±6.7 | 29.69±12.04 | 30.13±8.23 |
| **Proline** | 253.59±34.11 | 320.52±55.64* | 263.41±61.5# |
| **Pseudouridine** | 2.91±4.56 | 1.71±0.81 | 1.56±0.58 |
| **Serine** | 29.66±5.19 | 30.56±7.12 | 29.8±6.83 |
| **Serotonin** | 7.56±3 | 8.44±2.89 | 5.33±1.49# |
| **T-H-proline** | 54.74±50.28 | 86.92±72.16 | 64.91±49.75 |
| **Threonine** | 38.57±6.36 | 40.46±9.18 | 41.25±8.09 |
| **Tryptophan** | 14.9±5.9 | 17.62±3.04 | 15.82±4.72 |
| **Tyrosine** | 85.14±18.22 | 96.03±25.42 | 82.51±37.62 |
| **Urea** | 1347.46±415 | 1340.1±375.59 | 1165.51±249.99 |
| **Valine** | 62.64±9.72 | 95.62±19.45** | 65.15±24.63## |

For each group, n=6-8. * *P*<0.05 and ** *P*<0.01 vs. the Ctrl group. # *P*<0.05 and ##*P*<0.01 vs. the STZ group.

**Table S8**

**Metabolites corrected by-total protein and tryptophan indole D5 in T2DM mice.**

| **Metabolite** | **T2D-ctrl (ng/mg/ug/ml)** | **db/db**  **(ng/mg/ug/ml)** | **db+Art (ng/mg/ug/ml)** |
| --- | --- | --- | --- |
| **5-Hydroxytryptamine** | 0.06±0.01 | 0.06±0.03 | 0.04±0.02 |
| **AA** | 0.01±0 | 0.01±0.01 | 0.01±0 |
| **Alanine** | 128.91±32.34 | 145.18±53.81 | 153.22±19.13 |
| **Arginine** | 36.26±11.41 | 32.86±11.95 | 26.81±9.68 |
| **Asparagine** | 10.49±1.81 | 13.74±2.79 | 11.52±1.27 |
| **Aspartic acid** | 161.88±35.1 | 91.92±36.21** | 112.67±27.43 |
| **Citrulline** | 1.79±0.63 | 3.86±1.79 | 1.8±0.44 |
| **Cysteine** | 29.82±20.47 | 36.17±20.06 | 18.27±8# |
| **Glutamic acid** | 462.53±78.59 | 454.97±121.58 | 336.92±63.8# |
| **Glycine** | 196.27±45.3 | 110.49±26.83** | 83.84±16.65 |
| **Histidine** | 26.55±7.92 | 24.25±5.4 | 21.62±4.44 |
| **HVA** | 105.54±36.32 | 108.1±28.65 | 98.78±28.98 |
| **Isoleucine** | 14.24±3.2 | 19.7±5.38* | 15.07±4.19 |
| **KYA** | 0.07±0.04 | 0.19±0.27 | 0.05±0.02 |
| **Leucine** | 105.66±39.46 | 112.43±37.32 | 76.54±19.11 |
| **Lysine** | 21.04±5.93 | 17.59±2.44 | 14.25±1.64 |
| **MPT** | 0.37±0.14 | 0.77±0.7 | 0.35±0.09 |
| **Ornithine** | 5.75±1.13 | 5.64±1.68 | 4.32±1.04 |
| **Phenylanaline** | 36.56±13.43 | 46.99±12.75 | 39.35±10.38 |
| **Proline** | 274.89±71.8 | 296.97±87.53 | 250.43±29.93 |
| **Serine** | 30.97±8.33 | 22.14±5.31* | 21.8±3.5 |
| **Serotonin** | 6.67±2 | 4.56±1.05* | 2.74±1.13 |
| **Succinyladenosine** | 1.56±0.39 | 3.94±2.27** | 2.24±0.61# |
| **T-H-proline** | 14.69±4.1 | 19.74±5.6* | 14.6±3.63 |
| **Threonine** | 27.47±5.85 | 25.4±5.7 | 25.03±3.47 |
| **Tryptophan** | 14.66±4.67 | 14.94±3.5 | 12.99±2.61 |
| **Tyrosine** | 59.11±22.08 | 53.67±15.94 | 49.71±11.63 |
| **UA** | 6.66±1.27 | 9.93±5.21 | 9.76±3.3 |
| **Valine** | 53.18±15.63 | 65.25±22.16 | 50.39±10.96 |

For each group, n=6-8. * *P*<0.05 and ** *P*<0.01 vs. the Ctrl group. # *P*<0.05 vs. the db/db group.
